# Social and sexual consequences of facial femininity in a non-human primate

**DOI:** 10.1016/j.isci.2023.107901

**Published:** 2023-09-12

**Authors:** Sonia Tieo, Jules Dezeure, Anna Cryer, Pascal Lepou, Marie J.E. Charpentier, Julien P. Renoult

**Affiliations:** 1 CEFE, University Montpellier, CNRS, EPHE, IRD, Montpellier, France; 2 Projet Mandrillus, Fondation Lékédi pour la Biodiversité, Bakoumba BP 52, Gabon; 3 Institut des Sciences de l’Evolution de Montpellier (ISEM), UMR5554 - University of Montpellier/CNRS/IRD/EPHE, Place Eugène Bataillon, 34095 Montpellier Cedex 5, France

**Keywords:** Ethology, Biology of gender, Artificial intelligence

## Abstract

In humans, femininity shapes women’s interactions with both genders, but its influence on animals remains unknown. Using 10 years of data on a wild primate, we developed an artificial intelligence-based method to estimate facial femininity from naturalistic portraits. Our method explains up to 30% of the variance in perceived femininity in humans, competing with classical methods using standardized pictures taken under laboratory conditions. We then showed that femininity estimated on 95 female mandrills significantly correlated with various socio-sexual behaviors. Unexpectedly, less feminine female mandrills were approached and aggressed more frequently by both sexes and received more male copulations, suggesting a positive valuation of masculinity attributes rather than a perception bias. This study contributes to understand the role of femininity on animal’s sociality and offers a framework for non-invasive research on visual communication in behavioral ecology.

## Highlights

• AI-based method to estimate facial femininity
• 10k female mandrill images of 95 adult female mandrills analyzed
• Less feminine mandrills attracted more social and sexual attention

## Graphical abstract

**Figure:**
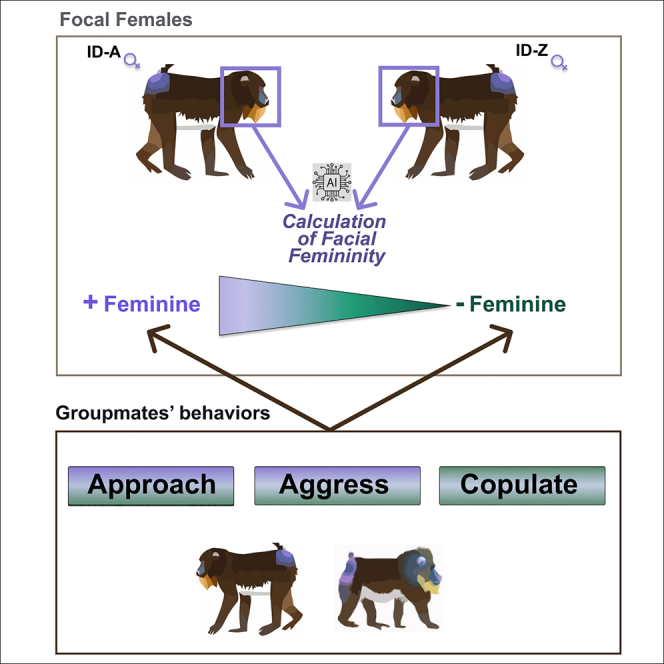

## Introduction

In humans, an attractive face confers various benefits ranging from having more sexual partners^1^ to being more successful at work,^2^ earning more money,^2^^,^^3^ and even receiving more lenient sentences in court.^4^ In women, facial femininity,^5^^,^^6^^,^^7^ the property of being representative of all female faces,^5^^,^^6^^,^^7^ is an important feature influencing attractiveness. Feminine faces are generally preferred over masculine faces in mating contexts,^5^^,^^6^^,^^7^ presumably because femininity is generally perceived as an indicator of fertility^8^ or maternal competencies.^9^ In various social contexts, both sexes also tend to prefer women with feminine traits, often associated with warmth, compared to masculine traits, generally associated with dominance and competitive tendencies.^10^^,^^11^^,^^12^ The influence of facial femininity on conspecific behavior and socio-sexual interactions remains, however, largely unexplored in other animal species, making it unclear whether these effects are unique to humans.^1^

Previous studies on captive macaques have shown that individuals can assess the degree of sexual dimorphism, one proxy of femininity, in conspecifics’ faces.^13^^,^^14^ However, femininity is only one of the multiple components influencing conspecifics’ behaviors. Indeed, both in humans^15^ and animals,^16^ behaviors emerge from multiple cognitive components, such as assessment (the quantification of a given value, e.g., attractiveness, associated with a stimulus), preference (the ranking of stimuli based on the assessment of different values), and choice (the selection of one stimulus based on multiple assessments, preferences, and other factors such as motor skills), which can be aligned or act in opposite directions.^16^ For example, both homosexual and heterosexual men show opposite sexual preferences despite similarly assessing the attractiveness of women’s faces,^17^ and men’s mate choice is modulated by contextual factors, such as the self-assessment of their own attractiveness (men who rate themselves as being more attractive generally choose more feminine women compared to men who rate themselves as being less attractive^18^^,^^19^). A large-scale cohort study found, moreover, that very attractive women (highest quartile) have more children than unattractive women, but fewer than moderately attractive women (second highest quartile),^20^ illustrating the complexity of mate choice decisions. Last, femininity can be evaluated differently in sexual contexts compared to social contexts. Although females’ facial femininity seems consistently positively correlated with attractiveness in a sexual context,^5^^,^^21^ in a social context, feminine women are often perceived as less competent, being possibly at a disadvantage compared to less feminine women.^10^^,^^22^^,^^23^ The valence of femininity, corresponding to a positive or a negative assessment, depends thus on the nature of the interactions in humans. Whether or not such valence can also switch depending on the context in other animal species with a rich social life remains unknown.

These studies were, however, designed to in-lab assessment of human facial attractiveness and femininity, possibly explaining the discrepancies observed. They thus provide probably limited information about the impact of femininity on actual social relationships observed in the wild. It is now necessary to study animal behaviors expressed in their natural environments. The study of femininity in wild animals raises, however, significant methodological challenges. In humans, the classical approach for quantifying facial femininity is based on highly standardized photographic portraits on which landmarks are positioned to align faces.^24^^,^^25^ Facial features are then extracted and used to build a face space, a multidimensional space that organizes variation among faces such that the distance between two faces describes their dissimilarity as perceived by humans.^26^^,^^27^ In a face space, the axis connecting the centroid of female faces to the centroid of male faces represents the axis of variation in femininity.^5^^,^^28^ In wild animals, obtaining standardized high-quality pictures is challenging. While captured and anesthetized individuals often show facial expressions that differ from their natural expressions, portrait images of freely moving animals are not standardized, showing high within-individual variation in pose, illumination, and facial expression that prevents the use of any landmark-based quantification methods. Fortunately, artificial intelligence (AI) now allows us to overcome these limitations. In particular, deep convolutional neural networks (DCNNs), the most widely used tool of AI in computer vision, automatically learn abstract features (e.g., colors, edges, simple and more complex textures) and concepts of interest (e.g., objects or parts of objects) when trained to optimally perform a specific task (e.g., individual recognition) from raw image data. Crucially, during training, DCNNs also learn to be insensitive to variations that are non-informative for that task (e.g., differences in illumination or distance to the camera in the case of individual recognition).^29^^,^^30^ Overall, DCNNs are powerful tools to build a face space in which individuals' position varies only with biologically relevant variables, such as identity, sex, or age.^29^^,^^31^

Here, we use DCNNs and long-term behavioral observations to investigate how facial femininity of female mandrills (*Mandrillus sphinx*), a primate from Central Africa, influences socio-sexual interactions in a large natural population of ∼250 wild individuals, monitored daily since 2012. Mandrills are a good study system for this topic because they are a highly dimorphic primate, with males being about 3.5 times heavier than females and exhibiting bright red and blue nasal and genital colorations mostly absent in females.^32^ Mandrills live in large multimale-multifemale social groups where both sexes are sexually promiscuous and where females form well-differentiated social interactions, with kinship and dominance ties having strong impacts on social partner choice. Mandrills are also seasonal breeders,^33^ and females may overlap in their fertility period,^34^ resulting in occasional male-mate choice. Indeed, during the mating season, males—usually the alpha male—mate-guard cycling females, favoring high-ranking ones when several females are receptive at the same time.^34^

In this study, we first developed an AI-based measure of femininity applicable to field pictures. We benchmarked the method in humans using both standardized and non-standardized photographic portraits scored for femininity and attractiveness by human judges. We then applied this method to 13,000 photographic portraits of adult female mandrills collected in their natural environment during daily monitoring, to obtain an annual femininity score—quantified in two different ways—for each studied female. Finally, we assessed how females’ femininity scores influence socio-sexual interactions with their groupmates (male copulations, intra- and inter-sexual spatial proximity, aggression, and grooming), using a large behavioral database collected over 10 years on 39 adult males and 95 adult females.

## Results

### AI model of facial femininity in women

**Figure 1:**
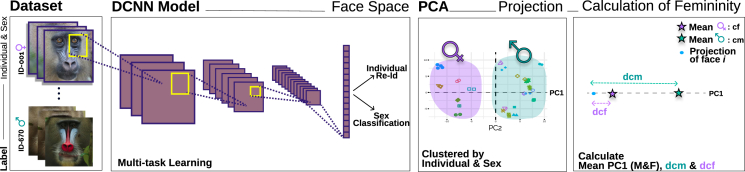
Pipeline to measure facial femininity in humans and mandrills DCNN model (VGGFace) was first retrained with FaceScrub database^37^ for humans and the Mandrillus Face Database^40^ for mandrills to simultaneously perform identity verification and sex classification (i.e., multi-task learning). Portrait pictures of the studied individuals were then fed to the newly trained DCNN, and their features extracted. The 128-d penultimate layer of the DCNN was used as a face space. Images of CFD database (for analyses on humans) and Mandrillus Face Database restricted to adults (for analyses on mandrills) were projected into their respective face space. Retrieved features were reduced further to two dimensions using a PCA.The third box (from the left) represents the position of different pictures of different individuals in the PC1-PC2 plane (for clarity, only twenty individuals are presented; one shape of a given color corresponds to one individual).While PC1 and PC2 together allowed us to cluster portraits collected on the same individual, PC1 alone discriminated the two sexes. We thus defined the female centroid, *cf*, and the male centroid, *cm*, as the mean PC1 score of all female and male pictures, respectively. We then calculated two measures of femininity for a given portrait picture *i*: (1) dcf, the distance between PC1_*i*_ and cf, which describes the averageness of a face within the female faces’ category, and (2) dcm, the distance between *PC*1_*i*_ and *cm*, which describes the dimorphism of females’ faces compared to males’ faces. In this depiction, a highly feminine face *i* is described by a high value of dcm_*i*_ and a small value of dcf.

We first developed a predictive model of facial femininity in women. We used publicly available datasets of human faces for which an empirical measure of femininity was obtained through psychological questionnaires. We then identified the measure of femininity obtained through DCNNs that best correlates with perceived femininity, that is, the score of femininity assessed by judges (Figure 1).

### Learning a face space

Estimating femininity requires first to build a face space. DCNNs can yield biologically realistic face spaces when trained to recognize individuals. Here, we used VGGFace,^35^ a DCNN^36^ previously trained to individually recognize more than two thousands celebrities from 2.6 million face images, as a base model for all analyses, for both humans and mandrills. Because VGGFace needs to be retrained with a mandrill portrait dataset to learn a mandrill face space (see in the following), for a comparable evaluation of model performance, we built a human face space by retraining VGGFace with another human face dataset (FaceScrub dataset^37^). Indeed, the quality of the retraining can be influenced by the size of the new dataset: we thus subsampled the FaceScrub dataset to keep the same number of individuals and mean number of images per individual as in our mandrill image dataset. Following previous studies,^27^^,^^29^ we used the penultimate layer of the retrained model as the face space. Indeed, with a DCNN trained to recognize individuals from their face, this layer encodes information about identity but also other characteristics of individuals, such as their sex and ethnic origin,^29^^,^^31^ while being relatively invariant to illumination, pose, and facial expression.^29^^,^^30^ We first retrained VGGFace to recognize individuals of the FaceScrub dataset using a classification task. The model achieved good performance (mean average precision mAP@3 accuracy: 97%); however, when examining the distribution of the faces within the face space, we noticed that female and male faces were largely overlapping (see Figure S1B in Supplementary Information). This contrasts with results in psychology showing that both sexes are likely represented in separate regions within humans’ perceptual face space.^29^^,^^30^ When training to recognize sex, faces collected on the same individual did not cluster together (Figure S1A), again challenging empirical findings in psychology.^30^ In order to build a face space that simultaneously separates sex and groups together portraits from the same individual (see Figure S1C), we thus retrained VGGFace to simultaneously recognize sex and verify individual identity. Face verification, the task of identifying whether two images depict the same individual or not, has been shown to better group together portraits collected on the same individual compared to a classification-based individual recognition.^38^ Our final model was able to recognize sex and verify identity with an accuracy of 99% and 91%, respectively.

### Measures of femininity in the face space

Studies on human faces traditionally investigate two measures of femininity: the femaleness (sexual dimorphism between male and female faces^39^) and the averageness within the female faces’ category.^28^ We evaluated the ability of both measures to predict facial femininity using the publicly available Chicago Face Dataset (CFD hereafter). We first projected every image of CFD onto our human face space and then reduced the dimensionality of this face space using a principal-component analysis (PCA). The first principal component (PC1) explained a relatively small fraction of the variance in features of the face space (15% for CFD); however, alone, it allowed us to separate the sexes with virtually no overlap (See Figure S2). Each face *i* was thus described by its PC1_***i***_ score. The female centroid, *cf,* and male centroid, *cm*, were computed as the mean of *PC1*_***i***_ score of all female and all male faces, respectively. We defined **“**averageness” within the female face category as dcf, the distance between *PC1*_***i***_ and *cf* and “femaleness” as dcm, the distance between *PC1*_***i***_ and *cm*, (see Figure 1). A high degree of averageness (low dcf) describes faces that are typical of the female category. We thus expect a negative correlation between dcf and perceived femininity; that is, a greater divergence from the average female face (increased dcf) is associated with a decreased perceived femininity. In contrast, faces exhibiting a higher degree of femaleness (high dcm) are perceived as sexually more dimorphic; we thus predict a positive correlation between dcm and perceived femininity.

**Figure 2:**
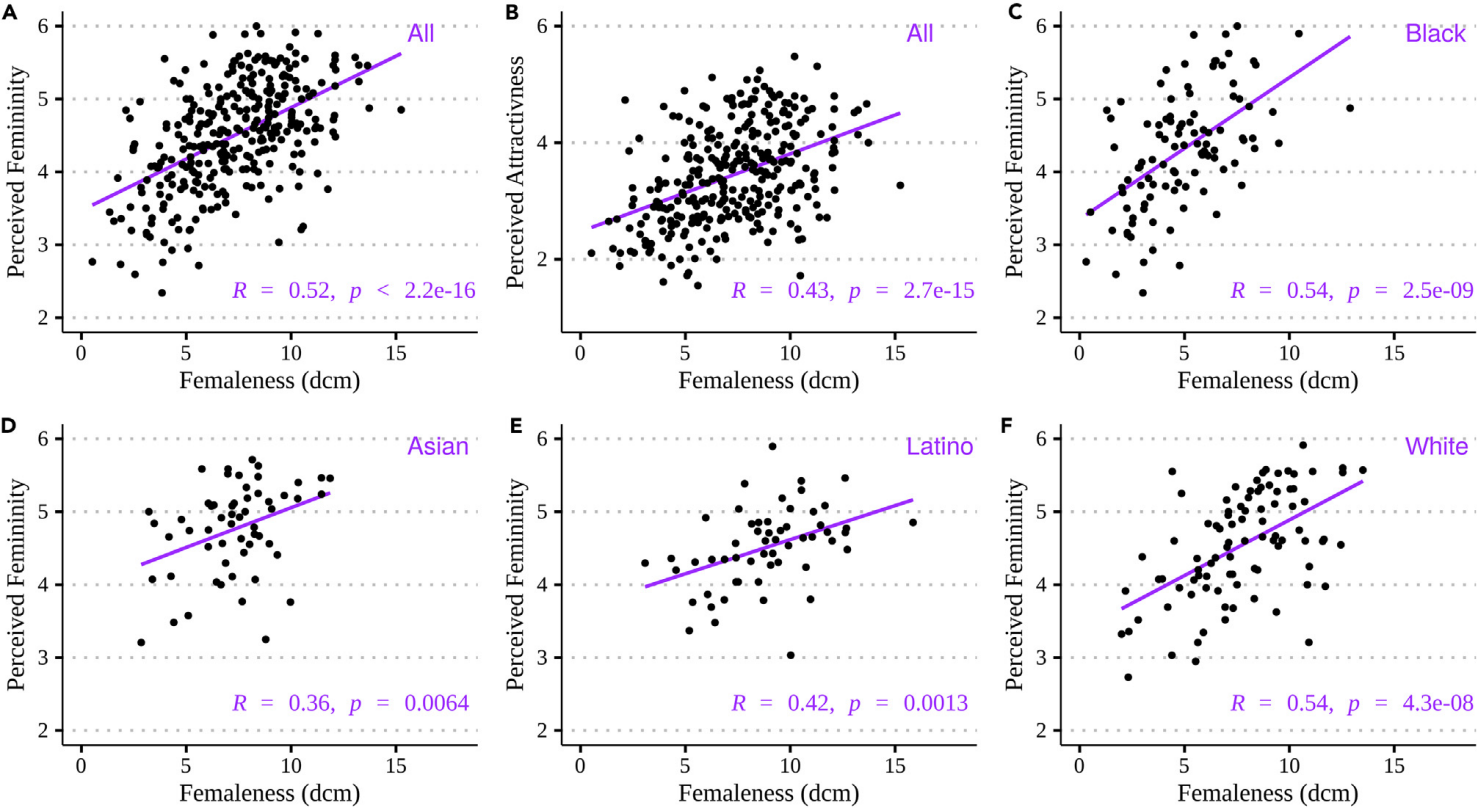
Correlation between predicted and perceived femininity/attractiveness in women (A–F) Results are shown for the Chicago Face Database, with predicted femininity measured as femaleness (dcm). Correlation with perceived femininity (A) and perceived attractiveness (B) for all 307 women, with perceived femininity for 104 Black (C), 57 Asian (D), 56 Latino, and 90 White females (F). R indicates the coefficient of correlation (Pearson) between predicted and perceived values, and *p* indicates the p value.

In accordance with our expectations, we found that dcm was positively (R = 0.52, p = 2.2e-16) and dcf was negatively (R = −0.16, p = 0.005) correlated with perceived femininity. Femaleness (dcm) was noticeably more strongly correlated with the perceived femininity of CFD than was averageness (dcf; Figure 2A). Because CFD includes faces of individuals from different ethnic backgrounds, we further calculated the correlation for each ethnic group. Across all ethnicities, we found, again, the highest correlations with dcm (Table S1) rather than dcf. The correlation was the highest for Black (R = 0.54, p = 2.5e-9; Figure 2C) and White (R = 0.54, p = 4.3e-8; Figure 2F) people, and the lowest for Asian people (R = 0.36, p = 0.0064; Figure 2D; see details in Table S1).

Most studies on facial femininity investigate the correlation between predicted femininity and perceived attractiveness (i.e., attractiveness rated by people), rather than perceived femininity. For example, Ryali and collaborators^41^ recently analyzed the CFD dataset using the widely used active appearance model^42^ of face space. They predicted femininity as statistical typicality (log likelihood of being a woman), a measure close (but inversely related) to our measure of averageness (dcf) when the probability density function is unimodal (as observed with CFD’s female face^41^), and they correlated their predicted femininity with the perceived attractiveness. They found a significant, positive correlation (R = 0.19) between the two measures, close in magnitude to the correlation between dcf and perceived femininity we found (R = −0.16). For a more accurate comparison, we also calculated the correlation between our predictions of femininity (dcf and dcm) and perceived attractiveness. As expected, with dcf, the correlation is significant (R = −0.14, p = 0.038) but slightly weaker than that with perceived femininity. With dcm, the correlation with perceived attractiveness is markedly stronger (R = 0.43, p = 2.7e-15) compared to what was previously reported.^41^

Last, we evaluated the performance of our method with non-standardized portrait pictures, as found in the mandrill portrait dataset but not in the CFD. To our knowledge, there is no publicly available dataset of non-standardized portraits associated with empirical scores of perceived femininity. We thus used SCUT-FBP5500,^43^ a dataset of non-standardized portraits retrieved from the Internet that display American and Asian people with faces of varying poses, expressions, and haircuts, with different illuminations and portrait backgrounds, and labeled for their perceived attractiveness. We found similar correlations between dcm and perceived attractiveness using either SCUT-FBP5500 (Asian: R = 0.33, p = 2.1e-51; Caucasian: R = 0.49, p = 1.1e-45) or CFD (Asian: R = 0.34, p = 0.0087; Caucasian: R = 0.47, p = 2.6e-6; see Tables S1 and S2).

Here, we thus showed that femaleness, i.e., the dimorphism of female faces compared to male faces, is a better predictor of femininity than the averageness within the category of female faces. We further showed that a face space automatically built from a DCNN trained to verify individual identity and discriminate sexes yields predictions of femininity with similar or higher performances compared to another, state-of-the-art face space. Crucially, however, with the DCNN-based face space, predictions are similarly good whether using in-lab, highly standardized portrait pictures, or non-standardized portraits of people in natural postures. Despite variations in performance, the method’s ability to explain part of femininity in all ethnic groups encourages us to apply it to non-standardized portraits of mandrills.

### Impact of femininity on mandrills' socio-sexual interactions

We estimated the femininity of adult females’ faces in mandrills, using the same methodology as developed in humans. We first learned a mandrill face space by retraining VGGFace to simultaneously classify the sex (accuracy after training: 99%) and verify the identity (accuracy after training: 60%) with a dataset of ca. 13,000 photographic portraits collected on 169 wild individuals. We then projected the images of all adult males and females into the face space, reduced the dimensionality of their features using a PCA, and eventually generated dcm values (i.e., the distance of a female face to the mean score of males along PC1; Figure 1) for each female as a measure of their femininity.

Contrary to the human datasets (CFD and SCUT-FBP5500), most study mandrills are represented by several pictures taken at different ages and in different conditions (maximum time lapse between two pictures of the same individual: 10 years), with a different number of pictures collected per individual (Figure S5). However, we found that the inter-individual variance in dcm (averaged over all pictures of a given individual) is 33.7, while the mean intra-individual variance is 1.4 (thus 23 times lower). In addition, the mean and variance in femininity scores are not correlated to the number of pictures considered (see STAR Methods and Figure S6). These results suggest that an estimate of femininity could be reliably obtained by simply averaging all images of a given individual. Nevertheless, in humans, the perception of femininity of a woman is influenced by age^44^ and possibly the menstrual cycle (^45^^,^^46^ but see Marcinkowska et al.^47^). We thus first investigated whether females’ age, dominance rank (high, medium, and low), sexual receptivity (whether she is cycling or not), and season (breeding vs. birthing) influence their facial femininity. To do so, we calculated one mean value of femininity per 6-month period for each of the 90 studied adult females (total: 5.5k pictures; mean ± SD: 263 ± 222 pictures per female and 140 ± 127 pictures per female per 6-month period). We matched these 6-month periods to relevant ecological characteristics of the social and environmental conditions experienced by the mandrills: each 6-month period roughly corresponds to either the birthing season (that mainly occurs during the long rainy season) or the breeding season (that mainly occurs during the long dry season). We found that only females’ age significantly correlates with dcm (estimate = −0.37, confidence interval [CI] = [-0.47 to −0.28], p value =<0.001; Table S3), with older females being less feminine than younger ones. This result is robust to various subsampling strategies accounting for potential spurious effects due to outliers (Figure S4). We thus used these femininity scores averaged over 6-month periods in the following behavioral analyses.

For each focal female, we analyzed the occurrence of different socio-sexual interactions with their male and female groupmates, each month of a given 6-month period: the monthly rate of copulations received from all adult males and the monthly rates of spatial association (time spent within 5 m), aggression, and grooming received from either all adult males or all other adult females. We performed one generalized linear mixed model per behavior, as the outcome variable, and considered the following explanatory variables: the female’s femininity score (dcm), her age and dominance rank, whether or not she was sexually receptive that month, and the season of behavioral sampling.

**Figure 3:**
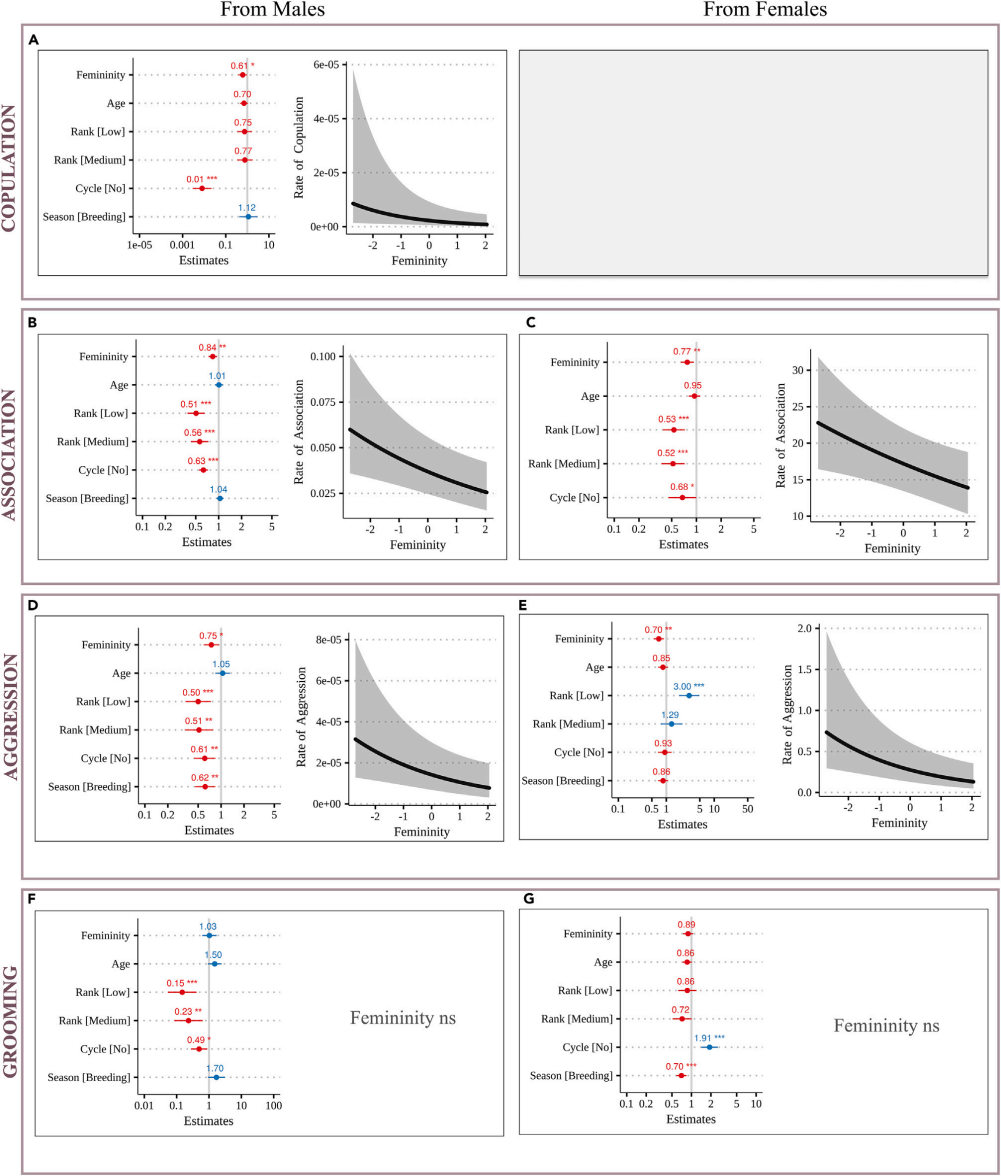
Predicted effect of femininity (dcm) on Mandrill Behavioral Rates Rates of copulation from males (A); rates of aggression from males (B) and females (C); rates of spatial association with males (D) and females (E); rates of grooming received from males (F) and females (G). Within each panel, the forest plot on the left gives the direction and size of the fixed effects on the outcome behavior. Significance is indicated (∗: p < 0.05, 0.01 and 0.001). The gray vertical line indicates no effect (here, 1 because it is a relative risk), and the horizontal bars indicate the confidence interval (95%). A dot on the right of the gray vertical line (>1, blue) indicates a positive correlation, whereas a dot to the left (<1, red) indicates a negative correlation. The predicted effect of dcm on the behavior is plotted on the right side of the panel, when significant. ns: not significant. Details in Tables S4–S12.

With respect to behaviors involving adult male groupmates, we found that males copulate more and are more associated with less feminine females (respectively: estimate = 0.61, 95% CI = [0.38–0.98], p = 0.041; estimate = 0.84, 95% CI = [0.74–0.95], p = 0.006) (Figures 3A and 3B; Tables S4 and S5). Furthermore, males aggress less feminine females significantly more than more feminine females (estimate = 0.75, 95% CI = [0.59–0.94], p = 0.013) (Figure 3D; Table S7). The effect of femininity on aggressions is partly explained by an increased spatial association of males with less feminine females, but not only. Indeed, the effect of femininity remains marginally significant (estimate = 0.82, 95% CI = [0.66–1.02], p = 0.070) when the rate of spatial association was included as a covariate in the model based on aggressions (Table S9). Last, femininity has no influence on grooming received by males (estimate = 1.03, 95% CI = [0.62–1.71], p = 0.908; Figure 3F; Table S11).

**Figure 4:**
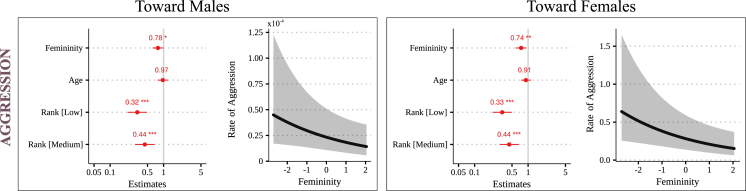
Predicted effect of femininity (dcm) on rates of aggression emitted toward males (left) and females (right) Within each panel, the forest plot on the left gives the direction and size of the fixed effects on the outcome behavior. Significance is indicated (∗: p < 0.05, 0.01 and 0.001). The gray vertical line indicates no effect (here, 1 because it is a relative risk), and the horizontal bars indicate the confidence interval (95%). A dot on the right of the gray vertical line (>1, blue) indicates a positive correlation, whereas a dot to the left (<1, red) indicates a negative correlation. The predicted effect of dcm on the behavior is plotted on the right side of the panel, when significant. Details in Tables S13 and S14.

With respect to behaviors involving other adult female group mates, we found that less feminine females are significantly more associated to their female groupmates (estimate = 0.90, 95% CI = [0.83–0.98], p = 0.014) (Figure 3C; Table S6) and also receive more aggression than more feminine females (estimate = 0.70, 95% CI = [0.54–0.89], p = 0.005) (Figure 3E; Table S8). This last effect remains marginally significant when accounting for spatial associations (estimate = 0.78, 95% CI = [0.61–1.00], p = 0.053; Table S10). Last, grooming relationships are not influenced by facial femininity of the study females (estimate = 0.89, 95% CI = [0.74–1.06], p = 0.197) (Figure 3G; Table S12).

We then asked whether facial femininity could be associated with behavioral traits expressed by the females. Specifically, we investigated if females displaying lower femininity give higher rates of aggression compared to more feminine females. We used a generalized linear mixed model with the female’s rate of emitted aggression as a response variable, and her femininity score (dcm), age, and dominance rank as explanatory variables. We found a significant negative association between femininity and the rate of emitted aggression. This association holds for both male (estimate = 0.75, 95% CI = [0.59–0.94], p = 0.013; Figure 4 and Table S14) and female targets (estimate = 0.75, 95% CI = [0.59–0.94], p = 0.013; Figure 4 and Table S15). Thus, females with lower facial femininity exhibit elevated rates of aggression.

## Discussion

In this study, we investigated whether facial femininity influences different socio-sexual interactions in a non-human primate. Using datasets with empirical scores of perceived femininity in humans, we first developed an AI-based method to predict femininity scores applicable to non-standardized pictures. We found that femaleness, a measure of sexual dimorphism, is more strongly correlated with perceived femininity than averageness, a measure of similarity to a mean female face. In mandrills, femaleness significantly influences several behaviors received from both male and female conspecifics, and this effect is consistent across various socio-sexual contexts, in all groupmates.

### Femininity influences socio-sexual behaviors in mandrills

Our results provide compelling evidence that socio-sexual partners behave differently toward female mandrills depending on the facial femininity of the target female. However, the direction of these effects raises questions about the nature of the information conveyed by facial femininity. Contrary to our expectations, we found that less feminine females, compared to more feminine ones, receive more copulations from males, are spatially closer to both male and female groupmates, and are also more frequently aggressed by them, independent of the females’ age, rank, sexual status, and the season of sampling. In this species, males are sexually coercive: they use long-term intimation through aggression toward females, which increases their future probability to mate with them once fertile.^48^ Aggressive male behaviors toward female mandrills therefore likely indicate sexual interest, although in this study we observed higher aggression toward less feminine females throughout the year, not only during the breeding season. The similar effect found for female aggressors, while controlling for female rank, could reflect intra-sexual competition. Female-female competition, compared to male-male competition, has received little scientific attention although reviews have highlighted that females may compete not only for food but also for access to mates or paternal care.^49^ If female mandrills compete for access to mates, and if males prefer less feminine females, then increased female aggression toward these preferred (less feminine) females is expected. The evolutionary reasons why females compete are outside the scope of this article, but, interestingly, intra-sexual competition mediated by facial femininity has also been suggested in humans, where women preferred by men are often envied by other women who resort to direct or indirect competition, such as the use of makeup or plastic surgery^50^ to rival them.^51^

Altogether, these results, in particular the increased rate of copulations from males toward less feminine females, may appear counterintuitive if femininity is a cue for females’ fertility and other maternal skills.^8^^,^^9^^,^^52^ However, the most feminine females are not always the most preferred sexual partners or the most feared sexual competitors, as has been observed in humans. For example, more feminine women are often judged less faithful and more promiscuous.^18^ Choosing feminine women would therefore entail the risk of being cheated on, and raising a child that is not one’s own.^18^ Moreover, high femininity in women can be associated with lower success in competition for resources and lower dominance, leading men to prefer women with more masculine faces, especially in challenging environments.^53^^,^^54^ These studies highlight the ubiquity of “flips” in the assignment of positive or negative valence to communicative traits.^55^ Flips in valence are not limited to humans though. In *Xiphophorus* swordtail fishes, for example, the general preference for pigmented caudal elongations has been reverted in some lineages.^56^^,^^57^ Observing a general preference for less feminine female faces in mandrills is thus not unexpected. A flip in the generally assumed valence of facial femininity would rather merely indicate that having masculine traits could be advantageous for female mandrills, e.g., to protect children against predators or to win resource conflicts. Interestingly, we found that less feminine females are more aggressive. This aggressiveness could drive the response of groupmates and thus partly explain that they are themselves more aggressive toward less feminine females. However, it is unlikely to explain all the differentiated behaviors expressed by groupmates, particularly the increase in copulations (in mandrills, females do not initiate copulations, pers. obs.). Since aggressiveness is generally determined by testosterone,^93^^,^^94^ this result rather reinforces the idea that a set of masculine behaviors could be selected behind the interest shown by males for females with less feminine faces. Future studies should explore this hypothesis, for example, by analyzing whether being a less feminine and more aggressive mother benefits their infants.

### The origin of the link between femininity and preference

A long-standing question in evolutionary psychology is whether the attractiveness of femininity results from specific adaptations to mate choice (i.e., the attribute indicator hypothesis), or whether it is rather a preference bias arising from general perceptual mechanisms.^58^^,^^59^ Our previous interpretation based on a flip in valence relies on an adaptive hypothesis: facial femininity influences preferences because it indicates other physical, physiological, or behavioral attributes (or lack thereof).^8^^,^^9^^,^^52^ Whether a preference bias is an alternative or a complementary explanation to our results, it is necessary to examine the relationship between femininity and femaleness first, and then those between femaleness and attractiveness.

Our result that femaleness in humans is a better predictor of femininity than averageness provides some support to the hypothesis that femininity is, at least partly, determined by perceptual mechanisms. This result probably reflects a contrast effect^60^ in which the perception of an individual stimulus is influenced by the statistical distribution of all stimuli in the category of the stimulus and also the distribution of stimuli in other categories. To our knowledge, only one study^61^ has previously compared how female facial averageness and sexual dimorphism predict perceived femininity. This study found that face shape dimorphism is correlated with femininity in two of the five ethnic groups studied (Colombian and Iranian), while averageness is correlated with femininity in two other groups (Czech and Turkish). The reason for the observed discrepancies across ethnicities in that study may be due to the fact their face space was based on morphometric features only, while our DCNN-based face space accounts for a greater diversity of features (see in the following). Beyond femininity, other studies have shown that dimorphism is a good predictor of the masculinity of male faces.^39^^,^^62^ In mandrills as well, the effects of a female’s femininity on the behaviors directed at her by both males and other females vanish when quantifying femininity as averageness (dcf) rather than femaleness (dcm; see Table S13). This result suggests that femaleness is also a better predictor of femininity in mandrills, and that a contrast effect probably occurs in this primate species too.

In our analyses of human faces, we further showed that femaleness is a good predictor of attractiveness. Again, this relationship could be interpreted through the lens of either the attribute indicator hypothesis or an incidental preference bias driven by perceptual mechanisms. The idea that the link between femininity and attractiveness is driven by perceptual mechanisms is rooted in the fluency theory of attractiveness^63^ and its vast empirical evidence.^64^^,^^65^ The fluency theory proposes that stimuli triggering a subjective sensation of ease in information processing are evaluated as being more attractive.^63^^,^^66^ Prototype-like stimuli are both easy to process (they are most quickly and precisely categorized and stored the longest in memory^67^^,^^68^) and are more attractive than non-prototype stimuli.^66^ The attractiveness of prototypes has been shown in humans, using various biological, inanimate, or even abstract visual stimuli without any indicated attributes.^66^^,^^67^ Regarding facial femininity, a study on chickens investigated preference for human faces: chickens trained to choose a typical female face from a range of male and female faces showed maximal response to female faces more distinctive than the mean during trials, specifically to the face that was also rated as the most attractive by human subjects.^69^ This result has been interpreted as evidence that facial femininity is attractive because it is easy on the mind.^70^ In mandrills, however, we found the opposite pattern: females with less feminine faces are spatially closer to groupmates, are more aggressed by groupmates, and receive more copulations from males than more feminine females. In this species, it therefore seems unlikely that the relationship between femininity and attractiveness is driven by fluency. This finding echoes classical critics of the fluency theory raised by researchers on artistic preferences, who stressed that attractive art can be perceptually challenging.^71^ Further studies in empirical aesthetics have shown that fluency effects actually amplify attractiveness when the stimulus already has a positive valence (i.e., is evaluated as beneficial), but the effect vanishes, or may even reinforce aversion,^72^ when the valence is flipped, as suspected in mandrills. To sum up, while we cannot rule out the possibility that femininity is a perceptual construct built from facial features only, our results highlight that in a non-human primate at least, the influence of femininity on the attractiveness of females is most likely driven by an adaptive, evaluative judgment, here a positive judgment of having masculine attributes.

### DCNN to study faces in the wild

Our method allowed us to explain 27% of the variance in perceived femininity ratings when considering all the studied ethnic groups and thus outperformed other face space-based methods for predicting femininity. In comparison, a previous study using a face space to calculate shape dimorphism in women explained a maximum of 20% of the variance in femininity.^61^ Another study^41^ used the active appearance model^42^ to construct a face space that takes into account both shape and texture information but not color. With this model, the authors explained 4% of the variance in attractiveness (these authors did not predict femininity) using the same dataset as the current study (CFD), compared to 18% of the variance in attractiveness with our method. As for studies on men, predictors of masculinity explain 15%^62^ to 25%^39^ of variance in perceived masculinity.

The transferability of the method to a non-human primate was encouraged by its ability to explain perceived femininity for all ethnic groups studied. Nevertheless, it is noteworthy that the performance varies substantially among ethnicities, ranging from 13% to 30% of variance. Unpublished investigations did not allow to identify the factors responsible for this variation in performance (particularly, the level of sexual dimorphism does not explain variation in performance, results not shown). Consequently, the actual ability of femaleness to predict perceived femininity by conspecifics outside the human species remains speculative.

Our high predictive performance in humans can probably be explained by the combination of our estimate of femininity (based on sexual dimorphism) and our use of a DCNN to define a face space rather than more classical methods. Indeed, a DCNN has several advantages for face studies. First, it can account for any features that are relevant to characterizing faces, including skin color and texture,^73^ the global shape, and the relative position of facial elements (mouth, nose, eye^74^). Second, with a DCNN, it is not necessary to predetermine and manually design supposedly relevant features: training the model to perform a high-level predictive task with these faces, for example, face recognition, is sufficient to learn and design features. Those features can be highly non-linear and more complex than handcrafted features.^31^^,^^75^ Interestingly, a DCNN trained for face recognition generates a representation that retains structured information about the face (e.g., identity, demographic characteristics, appearance, social features, expression), indicating that the nature of the training task is not critical to learn generalist facial features relevant to many communicative processes beyond face recognition.^29^^,^^31^ Nevertheless, in this study we showed that training a DCNN to perform both sex recognition and face verification, rather than face recognition, improves the prediction of femininity, indicating that it is worth adapting the learning task to a given research question (Figure S3). Third, DCNN-based face spaces are biologically realistic models of face representation. Many studies have highlighted similarities between DCNNs and the brain of primates in the way they process visual information and build internal representations of the world.^76^^,^^77^^,^^78^^,^^79^ Notably, DCNNs explain physical similarity^39^^,^^80^ and dissimilarity^27^ judgments between faces remarkably well. Lastly, DCNNs can be trained to be insensitive to the general coloration, brightness, and quality of an image, the pose and the emotion of a face, and its relative size on the image, if all these variations are non-informative for a given question (the case when studying femininity^29^^,^^31^). The invariance property of DCNNs explains that predictions of femininity in humans were similarly good with in-lab, standardized portrait images and with non-standardized images retrieved from the Internet. Faces are important for the visual communication of many animal species, playing a role for example in individual recognition, mate choice, kin discrimination, or social status advertising.^32^^,^^81^^,^^82^ Therefore, DCNNs provide new opportunities for non-invasive research in animal communication and behavioral ecology by enabling the investigation of the information conveyed through animals’ faces from portraits captured in their natural environments.

### Limitations of study

First, our methodology employs a DCNN, an AI-based approach, for predicting femininity scores from non-standardized images. While the DCNN presents inherent benefits, the adaptation of specific training tasks to our research question implies that further tailoring may be needed for application in other research contexts or species. Second, our study focuses on facial femininity and its impact on socio-sexual behaviors. This correlative approach does not allow, however, to explore the evolutionary mechanisms that drive the preference for less feminine facial features.

## STAR★Methods

### Key resources table

**Table:** 

REAGENT or RESOURCE	SOURCE	IDENTIFIER
**Deposited data**		

Mandrillus Face Dataset	Zenodo	https://doi.org/10.5281/zenodo.7467318
Cleaned Dataset	GitHub	https://github.com/soniamaitieo/Social_Sexual_Interactions_-Mandrills
Code	GitHub	https://github.com/soniamaitieo/Social_Sexual_Interactions_-Mandrills

**Experimental models: Organisms/strains**		

95 adult females (≥4 years) and 39 males (subadults or adults, ≥9 years) among free-ranging mandrills		

**Software and algorithms**		

R v3.6.3	Website	https://cran.r-project.org/
R packages are cataloged in a csv	GitHub	https://github.com/soniamaitieo/Social_Sexual_Interactions_-Mandrills
Python v3.8.5	Website	https://www.python.org/
Anaconda	Website	https://www.anaconda.com/
Python packages for the Anaconda environment are listed in a yaml file	GitHub	https://github.com/soniamaitieo/Social_Sexual_Interactions_-Mandrills

### Resource availability

#### Lead contact

Further information should be directed to and will be fulfilled by the lead contact, Sonia Tieo (tieo.soniamai@gmail.com).

#### Materials availability

This study did not generate new unique reagents.

### Experimental model and subject details

#### Ethical guidelines

The study received the necessary approval from the CENAREST institute (permit AR017/22/MESRSTTCA/CENAREST/CG/CST/CSAR), adhering to all relevant international, national, and institutional guidelines for the proper care and humane treatment of animals.

#### Study subjects

The study subjects are unfed and non-manipulated mandrills from a natural population living near the Lékédi Park in Southern Gabon. This group, which is fully habituated to humans, originated from captive mandrills that were released from the Center International de Recherches Médicales de Franceville (CIRMF) in 2002 and 2006.^88^ The group, daily monitored by the Mandrillus Project since its inception in 2012, comprised approximately 220 mandrills by the end of 2021, which included only 7 females that were born in captivity. For this study we considered 95 females who were 4 years of age or older and 39 males, either subadults or adults, aged 9 years and above.

### Method details

#### Image datasets

For retraining VGGFace to verify identity and classify sex, we used a subset of the FaceScrub dataset^37^ that includes 37,193 portrait images representing 530 individuals. Femininity and attractiveness analyses were performed with Chicago Face Database (CFD^83^) and SCUT-FBP5500 dataset.^43^ CFD provides standardized (neutral expression, frontal view, same clothes) photographic portraits of 597 American people (one photo per individual) aged 17 to 65 years, of different ethnic backgrounds (number of Women-Men; East Asian: 57-52, Black: 104-93; Hispanic: 56-52; White: 90–91). The face photographs were scored for attractiveness (from 1 to 6) and femininity (from 1 to 7) by independent judges. SCUT-FBP5500 includes 5,500 non-standardized portrait images of Asian (women: 2,000; men: 2,000) and White (women: 750; men: 750) people (one photo per individual) of different ages (15–60 years), and their associated scores of attractiveness (from 0 to 5) obtained by independent judges.

Mandrill portraits have been retrieved from the Mandrillus Face Database (MFD), which includes pictures collected between January 2012 and December 2021. The pictures were taken by field assistants directly in the forest, using different models of DSLR cameras and long-focal lenses, and different settings. Moreover, distance to the camera, illumination, pose and facial expression vary between pictures. Picture of MFD are thus qualified “non-standardized”. All images have been cropped manually to keep the face only (without the ear), and rotated to align the eyes horizontally. The quality of each image has been manually scored. Quality 1 corresponds to pictures of average quality for which experienced field assistants are able to recognize the individual from the picture alone. Quality 2 describes pictures of good quality for which individual recognition is straightforward but the portrait does not meet the criteria of quality 3. Quality 3 describes portrait pictures of maximal quality (“id card-like” portraits). For further information on image acquisition and pre-processing, see.^40^ For this study, we only considered adult individuals (females aged >4 years and males aged >9 years), corresponding to a total of 10,057 pictures collected on 119 adult females (83.8 ± 74 pictures per female) and 2,928 pictures from 50 adult males (57.4 ± 61.9 pictures per male). Femininity values (dcm and dcf) were obtained from these pictures on 119 females. Out of these 119 females, only 95 were used to determine their socio-sexual interactions with groupmates. Because the number of pictures per individual largely varies (Figure S5), we tested if this number influenced dcm and dcf when averaged over a 6-month period. We found no correlation between the number of pictures and both dcf and dcm values per individual, per 6-month period (Figure S6).

#### Face space

The face space was defined as the latent space (penultimate layer) of a customized and retrained version of VGGFace.^35^^,^^36^ After the second fully connected layer of the classical VGG16 architecture, we added a dropout layer (rate: 0.3), a dense layer with 128 neurons without activation (the Face Space), a lambda regularizer and a last dense layer with two neurons. The model was retrained to simultaneously verify the identity of individuals, using a Triplet Semi Hard loss function (from TensorFlow Addons^84^^,^^85^ connected to the face space, and to classify sex, using a sparse categorical cross entropy loss function connected to the 2-neuron dense layer. For training this model, we utilized 10,057 pictures with quality score ranging from 1 to 3. We used 60% of the images for training, 30% for validation and 10% for testing. Images were resized to 224x224 pixels. We used the Adam optimizer^86^ (learning rate: 0.001) and a batch size of 128. Model performances were evaluated on the test dataset using the mean average precision mAP@3 accuracy for face verification, and categorical accuracy for sex classification. Once retrained (i.e., performances did not improve further, usually after approx. 100 epochs), we used the model to encode new portrait images. For estimating femininity, we used pictures of quality scores 2 and 3 (thus not considering low-quality portraits). DCNN analyses were performed with Python 3 and TensorFlow.v2^87^ library on one NVIDIA GTX 1080 GPU.

### Quantification and statistical analysis

#### Calculation of femininity on the PC1 axis

To calculate dcf and dcm in mandrills, we used PC1 only. Indeed, we observed that the intra-individual variation of femininity is 23 times lower than the inter-individual variation when using PC1 only, while it is only 10 times or 14 times lower when using PC1-PC2 or the 128-day face space, respectively, because dimensions other than PC1 encode variations between faces that are not related to the sex. Female averageness (dcf), was calculated as the absolute distance between each individual face’s PC1 score (PC1i) and the PC1 projection of the female centroid (cf.; see also Figure 1):

*dcf = abs*(*PC1*_*i*_*- cf.*)

Similarly, femaleness (dcm) was calculated as the absolute distance between PC1i and the PC1 projection of the male centroid (cm):

*dcm = abs*(*PC1*_*i*_*- cm*)

Note that in our case (both in humans and mandrills) PC1_i_ - cm was always positive, meaning that using the absolute or the relative value yields similar results.

#### Mandrills’ behavioral data

Behavioral data were collected in the context of a long-term field project (the Mandrillus Project) that studies the socio-ecology of a natural population of mandrills habituated to human presence. The study population roams in a private park (the Lékédi Park) and its vicinity, close to the village of Bakoumba, in Southern Gabon (for more information, see^88^). Trained observers, blind to the study questions, perform daily 5-min focal sampling and *ad libitum* observations on all studied individuals.^89^ We used ∼13.019 h of focal data collected from 134 individuals: 95 adult females aged ≥4 years with 451 ± 739h of focals per female and 39 subadult and adult males aged ≥9 years with 514 ± 803h of focals per male (see details in Figure S7). During focals, all socio-sexual interactions are recorded between the focal individual and all individually-recognized social partners, including copulation (N = 132 events), grooming (N = 140 events), aggression (N = 656 events including chases, bites, ground-slapping; see^48^ for more information). Spatial associations are further recorded between the focal individual (1–3 scans per focal observation) and all groupmates located within a circle of 5m of radius length, (36,517 scans). Following Altmann’s observational research methods,^89^ we used a 5-meter radius scan, a standard method that allows for capturing comprehensive behavioral data. For the purpose of this study, we pooled all the focals performed on each study female with those collected on all other females or all other males present in the study group each month and for whom focals were available. For each female, we retrieved the number of monthly copulations received by all males, and the number of monthly grooming and aggressions received from all males and all females. We further retrieved the number of times all males or all females were found associated with each focal female using all scans performed on that female and her male and female partners, each month.

Birth dates of the study mandrills are either exactly known or estimated within a few days or months from daily monitoring or from general body condition and tooth wear patterns.^90^ Female dominance rank is estimated using outcomes of approach-avoidance collected during focal or *ad libitum* observations and calculated using standardized David’s score.^91^ Adult females are divided into three classes of rank of similar size over the study period (HR-high rank, MR-medium rank and LR-low rank) because female rank is stable in this matrilineal society.^92^

For each month of behavioral data collected on the focal females and their social partners, we determined whether these females were observed cycling that month (yes or no) thanks to a daily monitoring of female sexual cycles in the study population. Cycling females present conspicuous inflation of their genitalia, until they reach a maximum size corresponding to a fertile period that may last a few days.^33^

#### Statistical analyses

To evaluate the effect of femininity (dcm) on response variables (study behaviors), we used Generalized Linear Mixed Models (GLMM) with a negative binomial distribution and a logit function for over-dispersed count data. We log-transformed the total duration of focals, and the total number of scans performed, collected each month on each study female and all male or female groupmates and considered them as an offset (Nb_Scan_IndAllmal) in their respective models to control for different monthly duration of focals or scans performed. In addition to dcm calculated over 6-month periods corresponding to the breeding vs. the birthing season, we considered the following predictors in our statistical models as fixed effects: a female’s dominance rank (HR, MR or LR), age (years) and sexual status (cycling or not) at the month of behavioral data collection. Finally, we controlled for the season of sampling (breeding vs. birthing). We standardized all continuous predictors to compare estimates (dcm and age). The identity (id) of the focal female and the year of data collection were considered as two random effects to control for repeated monthly observations on focal females and yearly variation in sociality. For the rate of copulation, for example, the model is RateOfCopulation ∼ (1 | id) + (1 | year) + Femininity + Sexual status + Age + Dominance rank + offset(log(Nb_Scan_IndAllmal)). GLMMs were run using spAMM package with the *fitme* function (method = c("ML", "obs")). In preliminary analyses, we considered all possible first-order interactions involving dcm as well as the female age as a quadratic effect. None were significant, we thus removed these first-order interactions and the quadratic effect of age from final models. In order to represent behavioral rates, we first set the offset to 1 and then create a new data object; an argument to the pdep_effects function to evaluate the effect of a variable and then plotted this with ggplot2. The estimates are back transformed.

## Data Availability

• Data are publicly available as of the date of publication. Link is listed in the key resources table. • All original code has been deposited on GitHub and is publicly available as of the date of publication. The link is listed in the key resources table. • Any additional information required to reanalyze the data reported in this paper is available from the lead contact upon request.

## References

[1] Rhodes G., Simmons L.W., Peters M. (2005). Attractiveness and sexual behavior: Does attractiveness enhance mating success?. Evol. Hum. Behav..

[2] Hamermesh D.S. (2013).

[3] Biddle J.E., Hamermesh D.S. (1995).

[4] Ahola A.S., Christianson S.Å., Hellström Å. (2009). Justice needs a blindfold: Effects of gender and attractiveness on prison sentences and attributions of personal characteristics in a judicial process. Psychiatr. Psychol. Law.

[5] Perrett D.I., Lee K.J., Penton-Voak I., Rowland D., Yoshikawa S., Burt D.M., Henzi S.P., Castles D.L., Akamatsu S. (1998). Effects of sexual dimorphism on facial attractiveness. Nature.

[6] Rhodes G., Chan J., Zebrowitz L.A., Simmons L.W. (2003). Does sexual dimorphism in human faces signal health?. Proc. Biol. Sci..

[7] Fraccaro P.J., Feinberg D.R., DeBruine L.M., Little A.C., Watkins C.D., Jones B.C. (2010). Correlated male preferences for femininity in female faces and voices. Evol. Psychol..

[8] Smith M.J.L., Perrett D.I., Jones B.C., Cornwell R.E., Moore F.R., Feinberg D.R., Boothroyd L.G., Durrani S.J., Stirrat M.R., Whiten S. (2006). Facial appearance is a cue to oestrogen levels in women. Proc. Biol. Sci..

[9] Law Smith M.J., Deady D.K., Moore F.R., Jones B.C., Cornwell R.E., Stirrat M., Lawson J.F., Feinberg D.R., Perrett D.I. (2012). Maternal tendencies in women are associated with estrogen levels and facial femininity. Horm. Behav..

[10] Wen F., Zuo B., Ma S., Xu Y., Coley J.D., Wang Y. (2020). Do We See Masculine Faces as Competent and Feminine Faces as Warm? Effects of Sexual Dimorphism on Facial Perception. Evol. Psychol..

[11] Owen H.E., Halberstadt J., Carr E.W., Winkielman P. (2016). Johnny Depp, Reconsidered: How Category-Relative Processing Fluency Determines the Appeal of Gender Ambiguity. PLoS One.

[12] Neave N., Shields K. (2008). The effects of facial hair manipulation on female perceptions of attractiveness, masculinity, and dominance in male faces. Pers. Indiv. Differ..

[13] Rosenfield K.A., Semple S., Georgiev A.V., Maestripieri D., Higham J.P., Dubuc C. (2019). Experimental evidence that female rhesus macaques perceive variation in male facial masculinity. R. Soc. Open Sci..

[14] Damon F., Méary D., Quinn P.C., Lee K., Simpson E.A., Paukner A., Suomi S.J., Pascalis O. (2017). Preference for facial averageness: Evidence for a common mechanism in human and macaque infants. Sci. Rep..

[15] (2012). Evolution and the Mechanisms of Decision Making.

[16] Mendelson T.C., Fitzpatrick C.L., Hauber M.E., Pence C.H., Rodríguez R.L., Safran R.J., Stern C.A., Stevens J.R. (2016). Cognitive Phenotypes and the Evolution of Animal Decisions. Trends Ecol. Evol..

[17] Kranz F., Ishai A. (2006). Face perception is modulated by sexual preference. Curr. Biol..

[18] Little A.C., Jones B.C., Feinberg D.R., Perrett D.I. (2014). Men’s strategic preferences for femininity in female faces. Br. J. Psychol..

[19] Burriss R.P., Welling L.L., Puts D.A. (2011). Men’s attractiveness predicts their preference for female facial femininity when judging for short-term, but not long-term, partners. Pers. Indiv. Differ..

[20] Jokela M. (2009). Physical attractiveness and reproductive success in humans: Evidence from the late 20 century United States. Evol. Hum. Behav..

[21] O’Toole A.J., Deffenbacher K.A., Valentin D., McKee K., Huff D., Abdi H. (1998). The perception of face gender: the role of stimulus structure in recognition and classification. Mem. Cognit..

[22] Sczesny S., Spreemann S., Stahlberg D. (2006). Masculine = Competent? Physical Appearance and Sex as Sources of Gender-Stereotypic Attributions. Swiss J. Psychol. Schweiz. Z. Psychol. Rev. Suisse Psychol..

[23] Banchefsky S., Westfall J., Park B., Judd C.M. (2016). But You Don’t Look Like A Scientist!: Women Scientists with Feminine Appearance are Deemed Less Likely to be Scientists. Sex. Roles.

[24] Scheib J.E., Gangestad S.W., Thornhill R. (1999). Facial attractiveness, symmetry and cues of good genes. Proc. Biol. Sci..

[25] Jones B., Little A., Penton-Voak I., Tiddeman B., Burt D., Perrett D. (2001). Facial symmetry and judgements of apparent health: Support for a “good genes” explanation of the attractiveness–symmetry relationship. Evol. Hum. Behav..

[26] Tredoux C. (2002). A direct measure of facial similarity and its relation to human similarity perceptions. J. Exp. Psychol. Appl..

[27] Jozwik K.M., O’Keeffe J., Storrs K.R., Guo W., Golan T., Kriegeskorte N. (2022). Face dissimilarity judgments are predicted by representational distance in morphable and image-computable models. Proc. Natl. Acad. Sci. USA.

[28] Jones A., Jaeger B. (2019). Biological Bases of Beauty Revisited: The Effect of Symmetry, Averageness, and Sexual Dimorphism on Female Facial Attractiveness. Symmetry.

[29] Hill M.Q., Parde C.J., Castillo C.D., Colón Y.I., Ranjan R., Chen J.-C., Blanz V., O’Toole A.J. (2019). Deep convolutional neural networks in the face of caricature. Nat. Mach. Intell..

[30] Hopper W.J., Finklea K.M., Winkielman P., Huber D.E. (2014). Measuring sexual dimorphism with a race-gender face space. J. Exp. Psychol. Hum. Percept. Perform..

[31] O’Toole A.J., Castillo C.D., Parde C.J., Hill M.Q., Chellappa R. (2018). Face Space Representations in Deep Convolutional Neural Networks. Trends Cognit. Sci..

[32] Setchell J., Wickings E.J. (2005).

[33] Dezeure J., Charpentier M.J., Huchard E. (2022). Fitness effects of seasonal birth timing in a long-lived social primate living in the equatorial forest. Anim. Behav..

[34] Setchell J.M., Jean Wickings E. (2006). Mate Choice in Male Mandrills (Mandrillus sphinx). Ethology.

[35] Parkhi O., Vedaldi A., Zisserman A. (2015). BMVC 2015 - Proceedings of the British Machine Vision Conference 2015.

[36] Simonyan K., Zisserman A. (2015).

[37] Ng H., Winkler S. (2014).

[38] Gu J., Jiang W., Luo H., Yu H. (2021). An efficient global representation constrained by Angular Triplet loss for vehicle re-identification. Pattern Anal. Appl..

[39] Mitteroecker P., Windhager S., Müller G.B., Schaefer K. (2015). The morphometrics of “masculinity” in human faces. PLoS One.

[40] Tieo S., Restrepo-Ortiz C.X., Roura-Torres B., Sauvadet L., Harté M., Charpentier M.J.E., Renoult J.P. (2023). The Mandrillus Face Database: A portrait image database for individual and sex recognition, and age prediction in a non-human primate. Data Brief.

[41] Ryali C.K., Goffin S., Winkielman P., Yu A.J. (2020). From likely to likable: The role of statistical typicality in human social assessment of faces. Proc. Natl. Acad. Sci. USA.

[42] Cootes T.F., Edwards G.J., Taylor C.J. (1998). Active appearance models. Computer Vision —.

[43] SCUT-FBP5500: a diverse benchmark dataset for multi-paradigm facial beauty prediction. https://ieeexplore.ieee.org/abstract/document/8546038.

[44] Kloth N., Damm M., Schweinberger S.R., Wiese H. (2015). Aging affects sex categorization of male and female faces in opposite ways. Acta Psychol..

[45] Puts D.A., Bailey D.H., Cárdenas R.A., Burriss R.P., Welling L.L.M., Wheatley J.R., Dawood K. (2013). Women’s attractiveness changes with estradiol and progesterone across the ovulatory cycle. Horm. Behav..

[46] Roberts S.C., Havlicek J., Flegr J., Hruskova M., Little A.C., Jones B.C., Perrett D.I., Petrie M. (2004). Female facial attractiveness increases during the fertile phase of the menstrual cycle. Proc. Biol. Sci..

[47] Marcinkowska U.M., Holzleitner I.J. (2020). Stability of women’s facial shape throughout the menstrual cycle. Proc. Biol. Sci..

[48] Smit N., Baniel A., Roura-Torres B., Amblard-Rambert P., Charpentier M.J.E., Huchard E. (2022). Sexual coercion in a natural mandrill population. Peer Community J..

[49] Stockley P., Bro-Jørgensen J. (2011). Female competition and its evolutionary consequences in mammals. Biol. Rev..

[50] Nash R., Fieldman G., Hussey T., Lévêque J.L., Pineau P. (2006). Cosmetics: They Influence More Than Caucasian Female Facial Attractiveness. J. Appl. Soc. Psychol..

[51] Fink B., Klappauf D., Brewer G., Shackelford T.K. (2014). Female physical characteristics and intra-sexual competition in women. Pers. Indiv. Differ..

[52] Little A.C., Jones B.C., DeBruine L.M. (2011). Facial attractiveness: evolutionary based research. Philos. Trans. R. Soc. Lond. B Biol. Sci..

[53] Marcinkowska U.M., Kozlov M.V., Cai H., Contreras-Garduño J., Dixson B.J., Oana G.A., Kaminski G., Li N.P., Lyons M.T., Onyishi I.E. (2014). Cross-cultural variation in men’s preference for sexual dimorphism in women's faces. Biol. Lett..

[54] Little A.C., Cohen D.L., Jones B.C., Belsky J. (2006). Human preferences for facial masculinity change with relationship type and environmental harshness. Behav. Ecol. Sociobiol..

[55] Rosenthal G.G. (2018). Evaluation and hedonic value in mate choice. Curr. Zool..

[56] Basolo A.L. (1998). Evolutionary change in a receiver bias: a comparison of female preference functions. Proc. Biol. Sci..

[57] Wong B.B.M., Rosenthal G.G. (2006). Female Disdain for Swords in a Swordtail Fish. Am. Nat..

[58] Halberstadt J. (2006). The generality and ultimate origins of the attractiveness of prototypes. Pers. Soc. Psychol. Rev..

[59] Rhodes G. (2006). The evolutionary psychology of facial beauty. Annu. Rev. Psychol..

[60] Rosch E., Mervis C.B. (1975). Family resemblances: Studies in the internal structure of categories. Cognit. Psychol..

[61] Fiala V., Třebický V., Pazhoohi F., Leongómez J.D., Tureček P., Saribay S.A., Akoko R.M., Kleisner K. (2021). Facial attractiveness and preference of sexual dimorphism: A comparison across five populations. Evolut. Hum. Sci..

[62] Sanchez-Pages S., Rodriguez-Ruiz C., Turiegano E. (2014). Facial masculinity: how the choice of measurement method enables to detect its influence on behaviour. PLoS One.

[63] Reber R., Wurtz P., Zimmermann T.D. (2004). Exploring “fringe” consciousness: The subjective experience of perceptual fluency and its objective bases. Conscious. Cognit..

[64] Halberstadt J., Rhodes G. (2000). The attractiveness of nonface averages: implications for an evolutionary explanation of the attractiveness of average faces. Psychol. Sci..

[65] Rubenstein A.J., Kalakanis L., Langlois J.H. (1999). Infant preferences for attractive faces: a cognitive explanation. Dev. Psychol..

[66] (2003). The Hedonic Marking of Processing Fluency: Implications for Evaluative Judgment. The Psychology of Evaluation.

[67] Winkielman P., Halberstadt J., Fazendeiro T., Catty S. (2006). Prototypes are attractive because they are easy on the mind. Psychol. Sci..

[68] Whitfield T.W.A., Slatter P.E. (1979). The effects of categorization and prototypicality on aesthetic choice in a furniture selection task. Br. J. Psychol..

[69] Ghirlanda S., Jansson L., Enquist M. (2002). Chickens prefer beautiful humans. Hum. Nat..

[70] Renoult J.P., Mendelson T.C. (2019). Processing bias: extending sensory drive to include efficacy and efficiency in information processing. Proc. Biol. Sci..

[71] Muth C., Hesslinger V.M., Carbon C. (2015). The appeal of challenge in the perception of art: how ambiguity, solvability of ambiguity, and the opportunity for insight affect appreciation. Psychol. Aesthet. Creat. Arts.

[72] Albrecht S., Carbon C.-C. (2014). The Fluency Amplification Model: fluent stimuli show more intense but not evidently more positive evaluations. Acta Psychol..

[73] Kim Y., Hwang I., Cho N.I. (2017). 2017 IEEE International Conference on Image Processing (ICIP).

[74] Khan K., Attique M., Khan R.U., Syed I., Chung T.-S. (2020). A Multi-Task Framework for Facial Attributes Classification through End-to-End Face Parsing and Deep Convolutional Neural Networks. Sensors.

[75] Baek S., Song M., Jang J., Kim G., Paik S.-B. (2019). Spontaneous generation of face recognition in untrained deep neural networks. bioRxiv.

[76] Yamins D.L.K., Hong H., Cadieu C.F., Solomon E.A., Seibert D., DiCarlo J.J. (2014). Performance-optimized hierarchical models predict neural responses in higher visual cortex. Proc. Natl. Acad. Sci. USA.

[77] Kriegeskorte N. (2015). Deep Neural Networks: A New Framework for Modeling Biological Vision and Brain Information Processing. Annu. Rev. Vis. Sci..

[78] Raman R., Hosoya H. (2020). Convolutional neural networks explain tuning properties of anterior, but not middle, face-processing areas in macaque inferotemporal cortex. Commun. Biol..

[79] Lindsay G.W. (2021). Convolutional Neural Networks as a Model of the Visual System: Past, Present, and Future. J. Cognit. Neurosci..

[80] Jozwik K.M., Kriegeskorte N., Storrs K.R., Mur M. (2017). Deep Convolutional Neural Networks Outperform Feature-Based But Not Categorical Models in Explaining Object Similarity Judgments. Front. Psychol..

[81] Sheehan M.J., Tibbetts E.A. (2011). Specialized face learning is associated with individual recognition in paper wasps. Science.

[82] Charpentier M.J.E., Harté M., Poirotte C., de Bellefon J.M., Laubi B., Kappeler P.M., Renoult J.P. (2020). Same father, same face: Deep learning reveals selection for signaling kinship in a wild primate. Sci. Adv..

[83] Ma D.S., Correll J., Wittenbrink B. (2015). The Chicago face database: A free stimulus set of faces and norming data. Behav. Res. Methods.

[84] Schroff F., Kalenichenko D., Philbin J. (2015). 2015 IEEE Conference on Computer Vision and Pattern Recognition (CVPR).

[85] tfa.losses.TripletSemiHardLoss TensorFlow. https://www.tensorflow.org/addons/api_docs/python/tfa/losses/TripletSemiHardLoss.

[86] Kingma D.P., Ba J. (2014).

[87] Abadi M., Barham P., Chen J., Chen Z., Davis A., Dean J., Devin M., Ghemawat S., Irving G., Isard M. (2016).

[88] Peignot P., Charpentier M.J., Bout N., Bourry O., Massima U., Dosimont O., Terramorsi R., Wickings E.J. (2008). Learning from the first release project of captive-bred mandrills Mandrillus sphinx in Gabon. Oryx.

[89] Altmann J. (1974).

[90] Galbany J., Romero A., Mayo-Alesón M., Itsoma F., Gamarra B., Pérez-Pérez A., Willaume E., Kappeler P.M., Charpentier M.J.E. (2014). Age-related tooth wear differs between forest and savanna primates. PLoS One.

[91] Poirotte C., Massol F., Herbert A., Willaume E., Bomo P.M., Kappeler P.M., Charpentier M.J.E. (2017). Mandrills use olfaction to socially avoid parasitized conspecifics. Sci. Adv..

[92] Setchell J.M., Jean Wickings E. (2005). Dominance, Status Signals and Coloration in Male Mandrills (Mandrillus sphinx). Ethology.

[93] Archer J. (2006). Testosterone and human aggression: an evaluation of the challenge hypothesis. Neurosci. Biobehav. Rev..

[94] Batrinos M.L. (2012). Testosterone and aggressive behavior in man. Int. J. Endocrinol. Metabol..

