# Supplementary material for: Social and sexual consequences of facial femininity in a non-human primate
Source: iScience. 2023 Sep 12;26(10):107901. doi: 10.1016/j.isci.2023.107901 (PMC10520438; doi:10.1016/j.isci.2023.107901)
Supplement: Document S1. Figures S1–S7 and Tables S1–S15 [file mmc1.pdf]

iScience, Volume *26*

Supplemental information

Social and sexual consequences of facial femininity in a non-human primate

Sonia Tieo, Jules Dezeure, Anna Cryer, Pascal Lepou, Marie J.E. Charpentier, and Julien P. Renoult

Supplementary Information

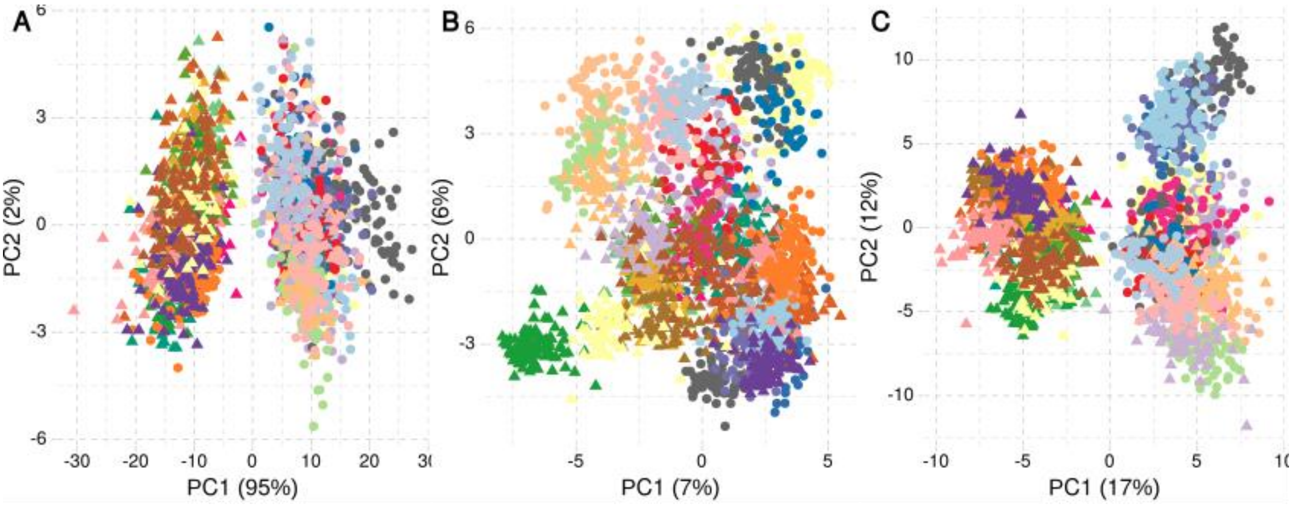

Figure S1. Influence of the learning task on the human face space, related to STAR Method. Human faces of FaceScrub dataset are projected into the face space, and their coordinates in the face space reduced by PCA. For visualization, coordinates along the first two principal components are shown for portraits of only 30 individuals colored by their identity. The face space corresponds to the 128-dimensional penultimate fully connected layer of VGGFace retrained on the FaceScrub dataset to perform different tasks: (A) sex classification, (B) individual verification, (C) individual verification and sex classification. This last task gives a face space that both separates sex and clusters same-individual portrait images. The percentages in the axis labels indicate the fraction of variance explained by PC1 and PC2. Triangles correspond to males, and circles to females.

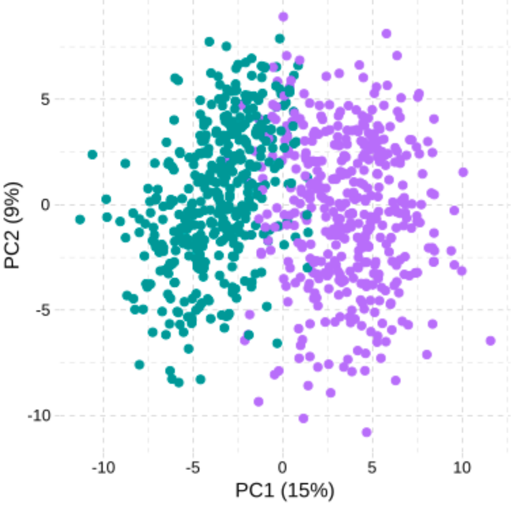

Figure S2. Visualization of the faces of the Chicago Face Dataset (CFD) into the PC1-PC2 plan of the face space, related to STAR Method. The portraits of the 597 individuals of CFD are colored by their sex (green for male and violet for female). The face space corresponds to the 128-dimensional penultimate fully connected layer of VGGFace retrained on the FaceScrub dataset to simultaneously perform identity verification and sex classification. Coordinates of CFD portraits in the face space were then reduced to two dimensions corresponding to the first two components (PC1 and PC2) of a PCA performed on the original 128 dimensions. The percentages in the axis labels indicate the fraction of variance explained by PC1 and PC2.

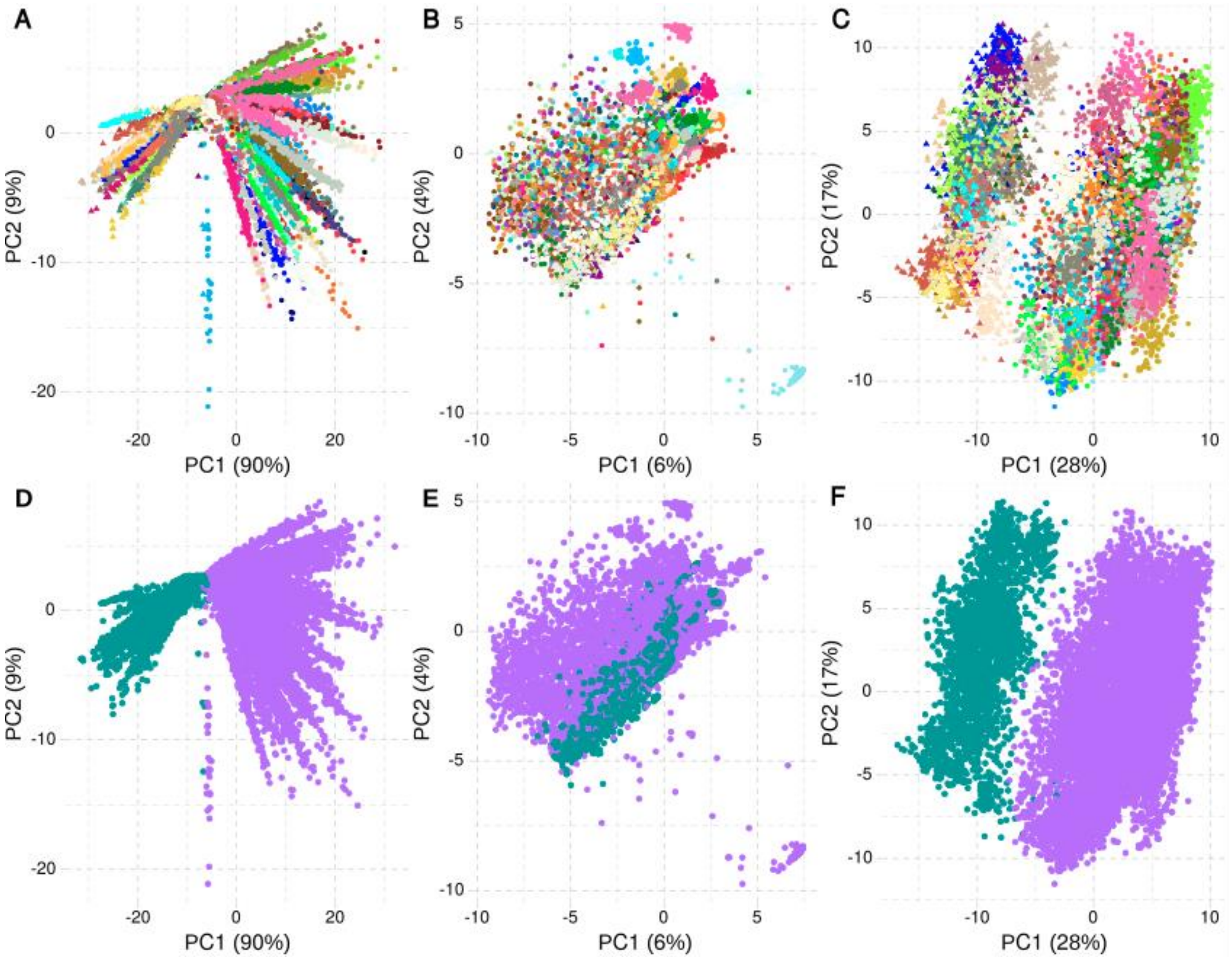

Figure S3. Influence of the learning task on the mandrill face space, related to STAR Method. The mandrill face space displays 12,985 pictures of 169 adult individuals from Mandrillus Face Dataset colored by their identity (A, B, C; triangles: males and circles: females) and their sex (D, E, F ; green: males and violet: females). We used the penultimate 128-dimensional fully connected layer of VGGFace retrained on the Mandrillus Face Dataset dataset as a face space. Portrait images of Mandrillus Face Dataset were then projected onto this face space, and their coordinates reduced by PCA. The percentages in the axis labels indicate the fraction of variance explained by PC1 and PC2. The projection on PC1 and PC2 gives different distributions of the faces depending on the learning task: (A,D) sex classification, (B,E) individual verification, and (D,F) individual verification plus sex classification. This last task gives a face space that both separates sex and clusters same-individual portrait images.

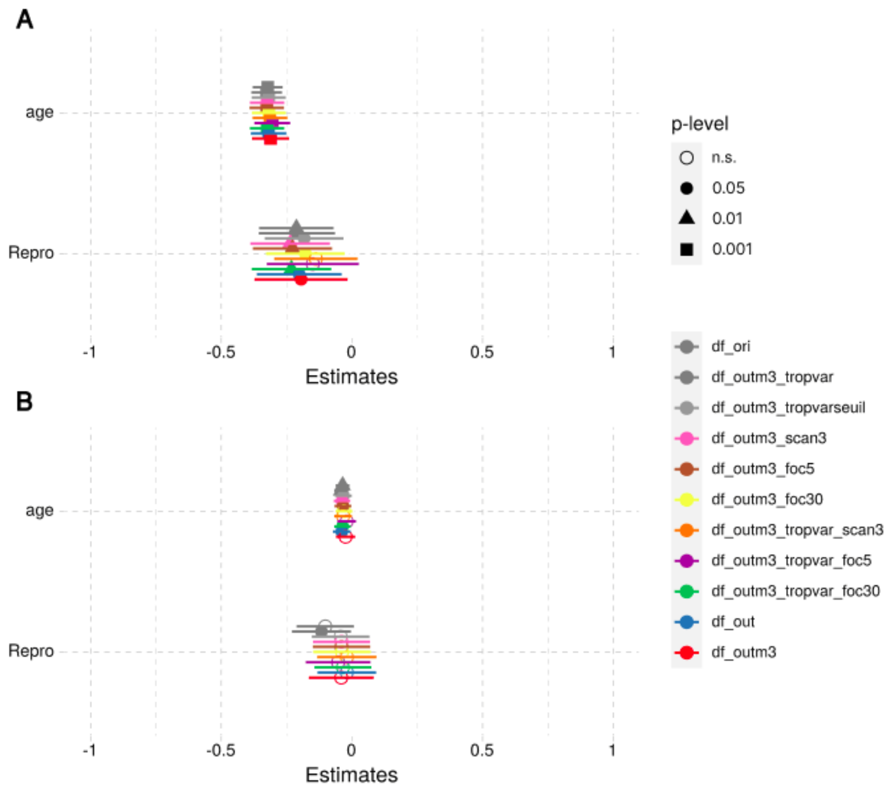

Figure S4. Effect of age and reproductive season on dcm (A) and dcf (B) on different subdataset, related to STAR Method. We fitted a linear mixed model (estimated using REML and nloptwrap optimizer) to predict dcm or dcf with age (continuous variable) and season (two-level categorical variable; formula: dcm ~ age + season). The model included the individual identity (id) and year of shooting (year) as random effects (formula: list(~1 | id, ~1 |year)). The forest plot of odds ratios gives the direction and size of the effects of the variables of interest (age and season). The symbols indicate the significance level and the error bar the standard error of the effect (see legend within the figure).Estimates equal to 0 indicate no effect. Dots on the left, negative side of zero shows a negative effect. These plots highlight the robustness of the model, that is, effect size and direction are similar across different strategies of data sampling: (1) df_ori: all observations included, (2) df_ot: photo outliers removed (based on IQR criteria), (3) df_outm3: photo outliers and observations with less than 3 pictures removed, (4) df_outm3_tropvar: photo outliers, observations with less than 3 pictures, and observations for which the intra-individual variance of dcm or dcf within a season is larger than the inter-individual variance removed, (5) df_outm3_tropvar: photo outliers, observations with less than 3 pictures, and observations for which the intra-individual variance of dcm or dcf within a season -with 10% tolerance- is larger than the inter-individual variance removed, (6) df_outm3_tropvar_scans3: photo outliers, observations with less than 3 pictures, observations for which the intra-individual variance of dcm or dcf within a season -with 10% tolerance- is larger than the inter-individual variance, and observations with less than 3 scans removed, (7) df_outm3_tropvar_foc5: photo outliers, observations with less than 3 pictures, observations for which the intra-individual variance of dcm or dcf within a season -with 10% tolerance- is larger than the inter-individual variance, and observations with less than 3 scans and with focal lengths of less than 5 minutes removed, (8) df_outm3_tropvar_foc30: photo outliers, observations with less than 3 pictures, observations for which the intra-individual variance of dcm or dcf within a season -with 10% tolerance- is larger than the inter-individual variance, and observations with less than 3 scans and with focal lengths of less than 5 minutes removed.

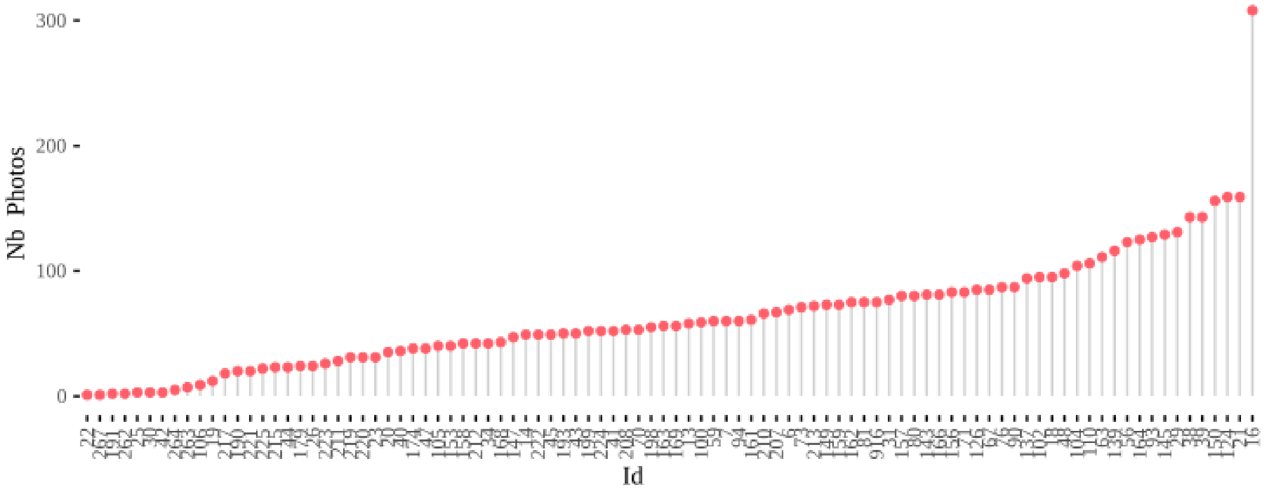

Figure S5. Number of photos per female of the studied mandrills population, related to STAR Method. Id along the x-axis indicates individual identifiers.

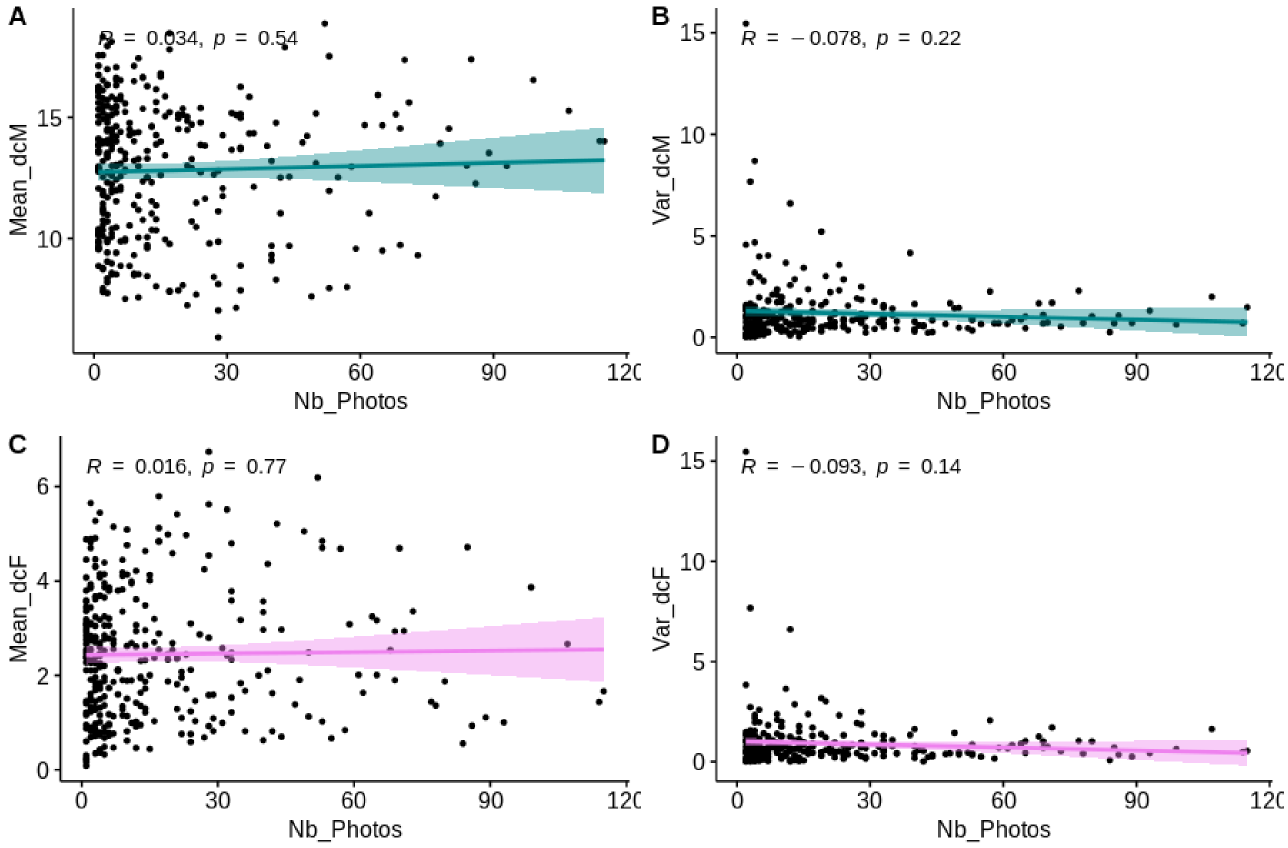

Figure S6. Correlation plot between mean or variance in femininity and the number of pictures, related to STAR Method. Observations are pooled by Y-S periods. Mean femininity (Mean_dcm or Mean_dcf) corresponds to the mean of dcm (or dcf) of all pictures of a female per Year-Season period, and Var_dcm (or Var_dcf) to the variance of dcm (or dcf) of all pictures of a female per Year-Season period. R corresponds to the Pearson coefficient of correlation and p to the p-value.

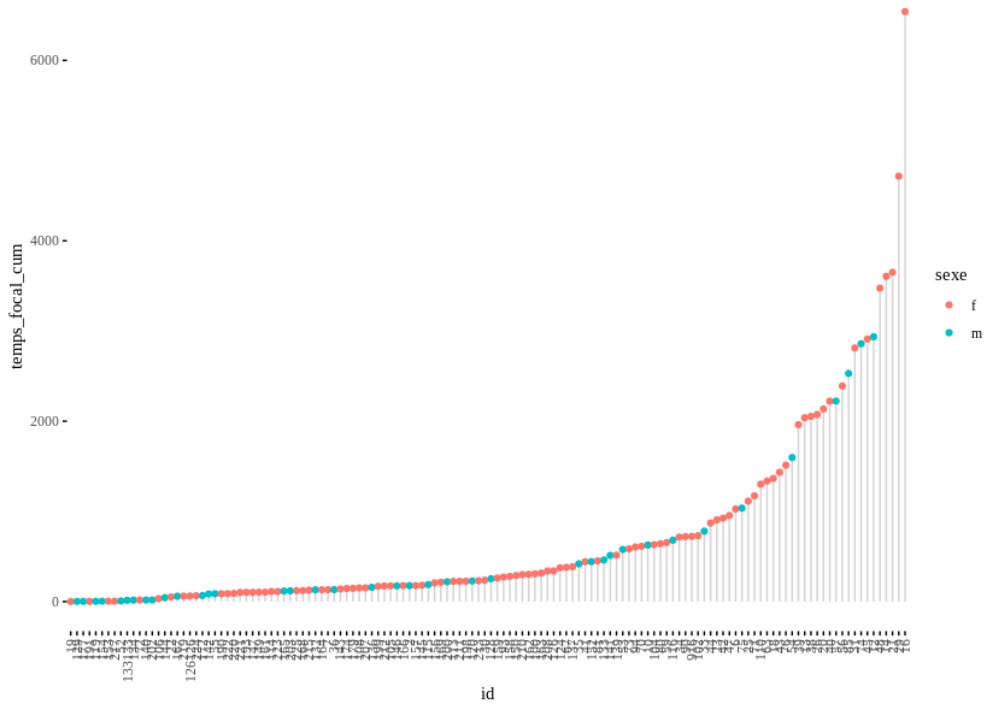

Figure S7. Cumulative focal times per individual. Id along the x-axis indicates individual identifiers.

Table S1. Correlation between the perceived femininity or attractiveness and the predicted femaleness or averageness for CFD dataset, related to STAR Method. Femaleness is calculated on PC1 (dcm) or in the 128d-FaceSpace (dcm_FaceSpace). Averageness is calculated on PC1 (dcf) or in the 128d-FaceSpace (dcf_FaceSpace). Separate analyses were conducted for all 307 females (All), 104 Black females, 57 Asian females, 56 Latino females, and 90 White females. R corresponds to the Pearson coefficient of correlation and p to the p-value.

CFD	**All**		**Asian**		**White**		**Black**		**Latino**	
	**R**	**p**	**R**	**p**	**R**	**p**	**R**	**p**	**R**	**p**
dcf_FaceSpace ~ Feminine	0.16	0.005	0.13	0.35	0.1	0.35	0.18	0.061	0.27	0.046
dcm_FaceSpace ~ Feminine	0.42	3e-14	0.24	0.07	0.39	0.00017	0.45	1.3e-06	0.4	0.0021
dcf ~Feminine	-0.16	0.005	-0.12	0.36	-0.2	0.058	-0.017	0.86	-0.11	0.42
dcm ~Feminine	0.52	1e-22	0.36	0.0064	0.54	4.3e-08	0.54	2.5e-09	0.42	0.0013
dcf_FaceSpace ~ Attractive	0.12	0.034	0.19	0.15	0.12	0.26	0.12	0.22	0.11	0.44
dcm_FaceSpace ~Attractive	0.33	2.8e-09	0.26	0.052	0.35	0.00061	0.36	0,0002	0.25	0.064
dcf ~Attractive	-0.12	0.04	-0.013	0.92	-0.14	0.17	0.047	0.63	-0.22	0.11
dcm ~ Attractive	0.43	2.7e-15	0.34	0.0087	0.47	2.6e-06	0.48	3.4e-07	0.3	0.025

Table S2. Correlation between the perceived attractiveness and the predicted femaleness or averageness for SCUT-FRB5000 dataset, related to STAR Method. Femaleness is calculated on PC1 (dcm) or in the 128d-FaceSpace (dcm_FaceSpace). Averageness is calculated on PC1 (dcf) or in the 128d-FaceSpace (dcf_FaceSpace). Separate analyses were conducted for all 2,750 females (All), 2,000 Asian females (Asian) and 750 White females. R corresponds to the Pearson coefficient of correlation and p to the p-value. The very low R score for dcm with All is due to that PC1 does not separate the sexes when all ethnicities are grouped together, since variation in ethnicities explains most of the variation between faces.

SCUT	**All**		**Asian**		**White**	
	**R**	**p**	**R**	**p**	**R**	**p**
dcf_FaceSpace ~ Attractive	0.098	2.6e-07	0.04	0.072	0.21	4.4e-09
dcm_FaceSpace ~Attractive	0.23	3.4e-34	0.19	2,00E-18	0.37	1.1e-25
dcf ~Attractive	-0.043	0.024	-0.013	0.57	0.061	0.092
dcm ~ Attractive	0.067	0.00042	0.33	2.1e-51	0.49	1.1e-45

Table S3. Effect of the season and female's age, dominance rank and sexual status on predicted femininity (dcm), related to STAR Method. We fitted a generalized linear mixed model (estimated using REML and nloptwrap optimizer) to predict dcm from the different predictors. Standardized parameters were obtained by fitting the model on a standardized version of the dataset. 95% Confidence Intervals (CIs) and p-values were computed using a Wald t-distribution approximation. Estimates are also provided. Significant effects are highlighted in bold. The mean value of dcm is 13 and the variance is 7.

Predictors	***Estimates***	***CI***	***p***
(Intercept)	0.02	-0.28 – 0.31	0.918
age	-0.37	-0.47 – -0.28	**<0.001**
saison repro [Repro]	0.01	-0.02 – 0.03	0.483
Cycle [NO]	0.01	-0.03 – 0.04	0.772
rank [LR] (ref: HR)	-0.08	-0.40 – 0.24	0.627
rank[MR] (ref: HR)	-0.01	-0.33 – 0.30	0.935
**Random Effects**			
σ2	0.07		
ICC	0.88		
N id	88		
N year	10		
Observations	2294		
Marginal R2 / Conditional R2	0.196 / 0.903		

Table S4. Model predicting the rate of copulation between a focal female and all males, related to Figure 3. We fitted a generalized linear mixed model (estimation of fixed effects by Laplace ML approximation (p_v.)) to predict the rate of copulation (number of events with the number of the observations as an offset; for predictions, the offset is set to 1). Formula: NbOfCopulation ~ (1 | id) + (1 | year) + Femininity + Cycle + Age + Rank + offset(log(Nb_Scan_IndAllmal)). The model included id and year as random effects (formula: list(~1 | id, ~1 |year)). The effect of femininity (dcm) is statistically significant and positive: males copulate more with less feminine females. Model performed with spaMM and results reported with sjPlot (R packages). Significant effects are highlighted in bold.

Predictors	***Incidence Rate Ratios***	***CI***	***p***
(Intercept)	0.00	0.00 – 0.00	**<0.001**
Femininity	0.61	0.38 – 0.98	**0.040**
Age	0.70	0.45 – 1.08	0.107
Rank [LR] (ref: HR)	0.75	0.34 – 1.65	0.471
Rank [MR] (ref: HR)	0.77	0.34 – 1.77	0.538
Cycle [No]	0.01	0.00 – 0.02	**<0.001**
Season [Breeding]	1.12	0.42 – 2.97	0.821
N id	88		
N year	10		
Observations	2239		

Table S5. Model predicting the rate of spatial association between a focal female and all males, related to Figure 3. We fitted a linear mixed model (estimation of fixed effects by Laplace ML approximation (p_v.)) to predict the rate of spatial association (number of events with the number of the observations as an offset; for predictions, the offset is set to 1). Formula: NbOfSA ~ (1 | id) + (1 | year) + Femininity + Cycle + Age + Rank + offset(log(Nb_Scan_IndAllmal)). The model included id and year as random effects (formula: list(~1 | id, ~1 |year)). The effect of femininity (dcm) is statistically significant and positive: males associate more with less feminine females. Model performed with spaMM and results reported with sjPlot (R packages).

Predictors	***Incidence Rate Ratios***	***CI***	***p***
(Intercept)	0.09	0.06 – 0.13	<0.001
Femininity	0.84	0.74 – 0.95	0.006
Age	01.01	0.89 – 1.14	0.905
Rank [LR] (ref: HR)	0.51	0.39 – 0.66	<0.001
Rank [MR] (ref: HR)	0.56	0.43 – 0.73	<0.001
Cycle [No]	0.63	0.55 – 0.72	<0.001
Season [Breeding]	01.04	0.94 – 1.16	0.436
N id	88		
N year	10		
Observations	2239		

Table S6. Model predicting the rate of spatial association between a focal female and all females, related to Figure 3. We fitted a linear mixed model (estimation of fixed effects by Laplace ML approximation (p_v.)) to predict the rate of Spatial Association (number of events with the number of the observations as an offset; for predictions, the offset is set to 1). Formula: NbOfSA ~ (1 | id) + (1 | year) + Femininity + Cycle + Age + Rank + offset(log(Nb_Scan_IndAllfem)). The model included id and year as random effects (formula: list(~1 | id, ~1 |year)). The effect of femininity is statistically significant and positive: females associate more with less feminine females. Model performed with spaMM and results reported with sjPlot (R packages).

Predictors	*Incidence Rate Ratios*	*CI*	*p*
(Intercept)	0.04	0.03 – 0.06	<0.001
Femininity	0.90	0.83 – 0.98	0.014
Age	0.94	0.86 – 1.03	0.172
Rank [LR] (ref: HR)	0.87	0.71 – 1.05	0.146
Rank [MR] (ref: HR)	0.76	0.62 – 0.93	0.006
Cycle [No]	1.09	0.99 – 1.19	0.073
Season [Breeding]	0.92	0.86 – 0.98	0.006
N id	88		
N year	10		
Observations	2239		

Table S7. Model predicting the rate of Aggression between a focal female and all males, related to Figure 3.. We fitted a linear mixed model (estimation of fixed effects by Laplace ML approximation (p_v.)) to predict the rate of Aggression (number of events with the number of the observations as an offset; for predictions, the offset is set to 1). Formula: NbOfAggression ~ (1 | id) + (1 | year) + Femininity + Cycle + Age + Rank + offset(log(Nb_Scan_IndAllfem)). The model included id and year as random effects (formula: list(~1 | id, ~1 |year)). The effect of femininity (dcm) is statistically significant and positive: males aggress more the less feminine females. Model performed with spaMM and results reported with sjPlot (R packages).

Predictors	*Incidence Rate Ratios*	*CI*	*p*
(Intercept)	0.00	0.00 – 0.00	<0.001
Femininity	0.75	0.59 – 0.94	0.013
Age	01.05	0.84 – 1.31	0.671
Rank [LR] (ref: HR)	0.50	0.34 – 0.74	<0.001
Rank [MR] (ref: HR)	0.51	0.33 – 0.79	0.002
Cycle [No]	0.61	0.44 – 0.84	0.003
Season [Breeding]	0.62	0.46 – 0.84	0.002
N id	88		
N year	10		
Observations	2239		

Table S8. Model predicting the rate of Aggression between a focal female and all females, ,related to Figure 3.. We fitted a linear mixed model (estimation of fixed effects by Laplace ML approximation (p_v.)) to predict the rate of Aggression (number of events with the number of the observations as an offset; for predictions, the offset is set to 1). Formula: NbOfAggression ~ (1 | id) + (1 | year) + Femininity + Cycle + Age + Rank + offset(log(Nb_Scan_IndAllfem)). The model included id and year as random effects (formula: list(~1 | id, ~1 |year)). The effect of femininity (dcm) is statistically significant and positive: females aggress more the less feminine females. Model performed with spaMM and results reported with sjPlot (R packages).

Predictors	*Incidence Rate Ratios*	*CI*	*p*
(Intercept)	0.00	0.00 – 0.00	<0.001
Femininity	0.70	0.54 – 0.89	0.005
Age	0.85	0.67 – 1.08	0.181
Rank [LR] (ref: HR)	3.00	1.84 – 4.88	<0.001
Rank [MR] (ref: HR)	1.29	0.76 – 2.18	0.341
Cycle [No]	0.93	0.67 – 1.29	0.671
Season [Breeding]	0.86	0.67 – 1.09	0.211
N id	88		
N year	10		
Observations	2239		

Table S9. Model predicting the rate of Aggression between a focal female and all males with Spatial Association as an explanatory variable, related to Figure 3. We fitted a linear mixed model (estimation of fixed effects by Laplace ML approximation (p_v.)) to predict the rate of Aggression (number of events with the number of the observations as an offset; for predictions, the offset is set to 1). Formula: NbOfAggression ~ (1 | id) + (1 | year) + Femininity + Cycle + Age + Rank + RateOfSA+ offset(log(Tps_Foc_IndAllmal)). The model included id and year as random effects (formula: list(~1 | id, ~1 |year)). The effect of femininity (dcm) is not statistically significant but positive. Model performed with spaMM and results reported with sjPlot (R packages). The effect of femininity on aggressions is partly explained by an increased spatial association of males with less feminine females, but not only. Indeed, the effect of femininity remains marginally significant (estimates = 0.82, 95% CI = [0.66 – 1.02], p = 0.070) when the rate of spatial associations was included as a covariate in the model predicting aggressions from femininity.

Predictors	*Incidence Rate Ratios*	*CI*	*p*
(Intercept)	0.00	0.00 – 0.00	<0.001
Femininity	0.82	0.66 – 1.02	0.070
Age	45078	0.87 – 1.30	0.542
Rank [LR] (ref: HR)	0.67	0.48 – 0.94	0.022
Rank [MR] (ref: HR)	0.64	0.43 – 0.97	0.036
Cycle [No]	0.70	0.50 – 0.98	0.039
Season [Breeding]	0.63	0.46 – 0.85	0.003
Rate of S.A.	857.37	85.24 – 8623.30	<0.001
N id	88		
N year	10		
Observations	2122		

Table S10. Model predicting the rate of Aggression between a focal female and all females with Spatial Association as an explanatory variable, related to Figure 3.. We fitted a linear mixed model (estimation of fixed effects by Laplace ML approximation (p_v.)) to predict the rate of Aggression (number of events with the number of the observations as an offset; for predictions, the offset is set to 1). Formula: NbOfAggression ~ (1 | id) + (1 | year) + Femininity + Cycle + Age + Rank + RateOfSA+ offset(log(Tps_Foc_IndAllfem)). The model included id and year as random effects (formula: list(~1 | id, ~1 |year)). The effect of femininity (dcm) is almost statistically significant and positive: females attack less feminine females. Model performed with spaMM and results reported with sjPlot (R packages). This effect remains marginally significant when accounting for spatial associations in the statistical model (estimates = 0.78, 95% CI = [0.61 – 1.00], p = 0.053; Table S10).

Predictors	*Incidence Rate Ratios*	*CI*	*p*
(Intercept)	0.00	0.00 – 0.00	<0.001
Femininity	0.78	0.61 – 1.00	0.053
Age	0.95	0.75 – 1.19	0.647
Rank [LR] (ref: HR)	3.25	2.03 – 5.19	<0.001
Rank [MR] (ref: HR)	1.47	0.88 – 2.45	0.140
Cycle [No]	0.92	0.65 – 1.30	0.646
Season [Breeding]	0.61	0.47 – 0.79	<0.001
Rate of S.A.	5.27	0.57 – 49.03	0.144
N id	88		
N year	10		
Observations	2122		

Table S11. Model predicting the rate of Grooming between a focal female and all males, related to Figure 3. We fitted a linear mixed model (estimation of fixed effects by Laplace ML approximation (p_v.)) to predict the rate of Grooming (number of events with the number of the observations as an offset; for predictions, the offset is set to 1). Formula: NbOfGrooming ~ (1 | id) + (1 | year) + Femininity + Cycle + Age + Rank + offset(log(Tps_Foc_IndAllmal)). The model included id and year as random effects (formula: list(~1 | id, ~1 |year)). The effect of femininity (dcm) is not statistically significant. Model performed with spaMM and results reported with sjPlot (R packages).

Predictors	*Incidence Rate Ratios*	*CI*	*p*
(Intercept)	0.00	0.00 – 0.00	<0.001
Femininity	1.03	0.62 – 1.71	0.908
Age	1.50	0.93 – 2.44	0.100
Rank [LR] (ref: HR)	0.15	0.05 – 0.41	<0.001
Rank [MR] (ref: HR)	0.23	0.08 – 0.64	0.005
Cycle [No]	0.49	0.28 – 0.89	0.018
Season [Breeding]	1.70	0.93 – 3.09	0.085
N id	88		
N year	10		
Observations	2122		

Table S12. Model predicting the rate of Grooming between a focal female and all females, related to Figure 3. We fitted a linear mixed model (estimation of fixed effects by Laplace ML approximation (p_v.)) to predict the rate of Grooming (number of events with the number of the observations as an offset; for predictions, the offset is set to 1). Formula: NbOfGrooming ~ (1 | id)++ (1 | year) + Femininity + Cycle + Age + Rank + offset(log(Tps_Foc_IndAllfem)). The model included id and year as random effects (formula: list(~1 | id, ~1 |year)). The effect of femininity (dcm) is not statistically significant. Model performed with spaMM and results reported with sjPlot (R packages).

Predictors	*Incidence Rate Ratios*	*CI*	*p*
(Intercept)	0.00	0.00 – 0.00	<0.001
Femininity	0.89	0.74 – 1.06	0.197
Age	0.86	0.73 – 1.02	0.075
Rank [LR] (ref: HR)	0.86	0.62 – 1.20	0.381
Rank [MR] (ref: HR)	0.72	0.51 – 1.01	0.054
Cycle [No]	1.91	1.42 – 2.56	<0.001
Season [Breeding]	0.70	0.58 – 0.84	<0.001
N id	88		
N year	10		
Observations	2122		

Table S13. Summary of dcm and dcf estimates with p-values for each behavior by males and females, related to Figure 3. Bold text indicates significant effects

		From Males	From Females
**dcm**	**MO**	**0.61 (p=0.040)**	
	**AG**	**0.75 (p=0.013)**	**0.70 (p=0.005)**
	**SCAN**	**0.84 (p=0.006)**	**0.90 (p=0.014)**
	**AG avec SCAN**	**0.82 (p=0.070)**	**0,78 (p=0.053)**
	**TO**	1.03 (p=0.908)	0.89 (p=0.197)
**dcf**	**MO**	1.21 (p=0.245)	
	**AG**	0.99 (p=0.896)	1.01 (p=0.890)
	**SCAN**	1.00 (p=0.934)	0.96 (p=0.134)
	**AG avec SCAN**	0.95 (p=0.467)	1.02 (p=0.797)
	**TO**	0.96 (p=0.823)	0.98 (p=0.779)

Table S14. Model predicting the rate of Aggression given by females with femininity score toward males, related to Figure 4. We fitted a linear mixed model (estimation of fixed effects by Laplace ML approximation (p_v.)) to predict the rate of Aggression (number of events with the number of the observations as an offset; for predictions, the offset is set to 1). Formula: NbOfAggression ~ (1 | id) + (1 | year) + Femininity + Age + Rank + offset(log(Nb_Scan_IndAllmal)). The model included id and year as random effects (formula: list(~1 | id, ~1 |year)). The effect of femininity (dcm) is statistically significant and positive: females aggress more females when they are less feminine. Model performed with spaMM and results reported with sjPlot (R packages).

Predictors	Incidence Rate Ratios	CI	p
(Intercept)	0.00	0.00 – 0.00	<0.001
Mean dcM	0.78	0.62 – 0.99	0.041
age	0.97	0.78 – 1.21	0.772
rang [LR] (ref: HR)	0.32	0.21 – 0.49	<0.001
rang [MR] (ref: HR)	0.44	0.29 – 0.67	<0.001
N id	88		
N year	10		
Observations	2239		

Table S15. Model predicting the rate of Aggression given by females with femininity score toward females, related to Figure 4. We fitted a linear mixed model (estimation of fixed effects by Laplace ML approximation (p_v.)) to predict the rate of Aggression (number of events with the number of the observations as an offset; for predictions, the offset is set to 1). Formula: NbOfAggression ~ (1 | id) + (1 | year) + Femininity + Age + Rank + offset(log(Nb_Scan_IndAllfem)). The model included id and year as random effects (formula: list(~1 | id, ~1 |year)). The effect of femininity (dcm) is statistically significant and positive: females aggress more males when they are less feminine. Model performed with spaMM and results reported with sjPlot (R packages).

Predictors	Incidence Rate Ratios	CI	p
(Intercept)	0.00	0.00 – 0.00	<0.001
Mean dcM	0.74	0.59 – 0.93	0.010
age	0.91	0.73 – 1.13	0.392
rang [LR] (ref: HR)	0.33	0.22 – 0.49	<0.001
rang [MR] (ref: HR)	0.44	0.29 – 0.67	<0.001
N id	88		
N year	10		
Observations	2239		
